# Force-sensing glove with real-time visual feedback for cello right-hand technique instruction

**DOI:** 10.3389/fpsyg.2026.1856906

**Published:** 2026-09-03

**Authors:** Diego Arbizu, Aimar Yeregui, Igor Saenz, Alicia Martínez-Ramírez, Montserrat Pàmies-Vilà

**Affiliations:** 1 Department of Human Sciences and Education, Public University of Navarre, Pamplona, Spain; 2 String Instruments Department, Higher Conservatory of Music of Navarre, Pamplona, Spain; 3 Department of Statistics, Computer Science and Mathematics, Public University of Navarre, Pamplona, Spain; 4 Department of Music Acoustics–Wiener Klangstil, mdw–University of Music and Performing Arts Vienna, Vienna, Austria

**Keywords:** bowing technique, cello pedagogy, force-sensing resistors, motor learning, music performance

## Abstract

**Introduction:**

Bow force and movement are intrinsic to learning and performing bowing techniques on bowed string instruments. This study explores the use of a low-cost sensorised glove designed to support right-hand technique learning in cello performance.

**Methods:**

The glove is fitted with piezoresistive sensors on the thumb and index finger to quantify grip force during bowing, enabling the evaluation of bow-hold ergonomics in real time. Tests with five cello students comprised three tasks (holding the bow still, sustaining a note, and performing a one-octave scale), followed by participation in a discussion group.

**Results:**

The results revealed consistent finger force patterns across all exercises, with higher forces occurring toward the bow tip and lower values near the frog, reflecting the natural lever dynamics of cello playing. Force behaviour proved stable and repeatable within the selected physiological range, confirming the glove's reliability for capturing meaningful variations in bowing technique despite reduced sensitivity at higher loads. Participants reported that the glove enhanced their awareness of applied pressure during *sostenuto*.

**Discussion:**

These findings suggest perceived pedagogical potential for self-monitoring and technique refinement while demonstrating the feasibility of using visual real-time wearables in instrument instruction. Future research could include longitudinal studies and the synchronisation of audio recordings to further explore the relationship between applied finger forces and sound production.

## 1 Introduction

Over the past centuries, bowing technique on string instruments has reached remarkable levels of virtuosity. Nevertheless, this progress in instrumental performance has not been accompanied by comparable developments in teaching methodologies, which still largely depend on the teacher's gestural demonstration and the oral transmission of experiential knowledge. The present study offers both qualitative and quantitative contributions towards establishing a novel tool designed to visualise bow control in pedagogical contexts, where the integration of technology into teaching and learning remains comparatively limited. In parallel, it advances the understanding of cello right-hand technique by providing measurable insights into the underlying physical and gestural parameters of bowing. Throughout this publication we refer to two parts of the bow: the frog, where the bow is held by the right hand, and the tip, the opposite extreme of the bow.

In recent years, a large number of studies have been conducted on bowed string instruments with the aim of analysing the bowing forces and the bow motion. Combining force sensors on the fingers of the bowing hand with audio, video and motion-recording software has many applications, including performance analysis, pedagogical purposes and personal instrumental study (Schoonderwaldt and Demoucron, 2009). Many studies concentrated on violin performance, particularly focusing on the analysing of complex movements and changes in finger force distribution. Großhauser et al. (2010) proposed practical applications including the recognition of inaccuracies, cramps, and incorrect postures, as well as the possible development of augmented instruments and new performance techniques. Similarly, Großhauser and Tröster (2014) worked on a solution based on force-sensitive resistors on the left hand to detect position and force between the contact points of the fingers and the musical instrument. The aim was to discreetly integrate sensors into a traditional musical instrument by constructing a sensorised violin fingerboard.

Conversely, motion analysis of various parameters related to bowing technique in string instruments has received increasing attention (Schoonderwaldt and Demoucron, 2009). This development has been supported by advances in motion capture technology and its application to artistic disciplines such as music performance. The most commonly used measurement methods include video recordings, infrared camera systems, optical tracking techniques, and the use of acceleration sensors and gyroscopes (Großhauser and Tröster, 2014). The application of 3D motion capture technology to the analysis of joint movements involved in bowing has helped to stabilise key performance parameters such as bow angle, speed, and acceleration (Schoonderwaldt and Demoucron, 2009; Maestre, 2009; Dalmazzo and Ramírez, 2019). These studies have highlighted the importance of the articulated use of the right arm and demonstrated that experienced musicians exhibit greater variability in bowing parameters (Verrel et al., 2014).

The development of systems that enable performers and students to visualise their gestures in real time during performance through motion capture techniques opens up extensive pedagogical possibilities for teaching and learning. For instance, Dalmazzo and Ramírez (2019) collected motion and audio data from a professional violinist performing seven bowing techniques, obtaining inertial motion data from the right forearm, synchronised with audio recordings. Similarly, Schoonderwaldt and Demoucron (2009) developed a method to quantify a series of bowing parameters in violin performance. Using optical motion capture cameras and force sensors, they achieved precise measurements of primary parameters (bow position, speed, acceleration, distance from the bridge, and applied force) as well as secondary ones (obliqueness, inclination, and angularity of the bow hair relative to the strings). They also identified additional performance aspects such as string contact (on- or off-string), the specific string being bowed, and bowing direction (down-bow or up-bow), all without interfering with the performer's playing. Along parallel lines, Guaus et al. (2007) addressed the challenge composers face in encoding certain parameters (e.g., bow speed, applied pressure, and fingering) into MIDI files, despite their crucial role in shaping sound and phrasing. They developed a sensing system using two strain gauges mounted at the frog and tip of a violin bow to capture force variations exerted by the player. A further example is the MusicJacket prototype developed by Van der Linden et al. (2011), a wearable motion-capture system designed as a pedagogical tool for violin instruction, focusing on body posture and bowing technique. The system uses inertial sensors to provide real-time feedback on the player's instrument hold and bow trajectory. Vibrotactile actuators on the arm and torso offer corrective feedback regarding bow movement and posture. In a comparative study involving beginner violinists, those using MusicJacket outperformed a control group receiving only traditional instruction, indicating that vibrotactile feedback effectively supports the acquisition of basic bowing skills. Following a similar line of research, Di Tocco et al. (2019) proposed a low-cost wearable system based on piezoresistive sensors integrated into elbow and wrist garments to monitor bowing movements, demonstrating its feasibility for identifying bow strokes and string changes while highlighting its potential as a quantitative support tool for beginners. More recently, Di Stefano et al. (2024) employed wearable inertial and physiological sensors to investigate the relationship between bowing kinematics, technical difficulty, and perceived musical expressivity in violinists. They found that movement smoothness was associated with musical expressivity, emphasising the value of objective biomechanical measurements towards understanding motor control and informing music education.

Recognising the importance of cello position and playing posture in instrumental learning, Scheiblauer et al. (2022) conducted a study with advanced cellists using optical motion capture technology to analyse their posture, cello position, and bowing movements. Later experiments incorporated a robotic arm programmed with bowing motion data, enabling replication of bow trajectories under controlled conditions (Pàmies-Vilà et al., 2024). In related work on bouncing bow strokes, Salvador-Castrillo et al. (2024) employed an experimental violin bow and used motion capture to examine the moment of inertia occurring during stick rotation in the *sautillé* technique. On a commercial level, the K-Bow project by Keith McMillen^1^ offers a sensor-based bow capable of measuring grip force, bow-hair force on the string, acceleration, and bow position. Together, these studies point to significant potential for advancing analytical and pedagogical methodologies in cello performance and instruction.

Comparable technological developments have also emerged in the study of woodwinds and brass instrument performance. As such, Hofmann and Goebl (2016) instrumented a Viennese clarinet with sensors to measure finger forces during performance. Their findings showed that clarinettists apply greater force when playing expressively than during technical exercises, indicating a measurable connection between physical interaction and musical expression. Similar attempts to integrate sensing technology into brass instruments are seen in the work of Amann et al. (2023), who investigated real-time acquisition of acoustic parameters at the embouchure of a trumpet or trombone by designing a digital, networked mouthpiece. The proposed system fits the microelectronic components and sensors at the mouthpiece, and it is capable of wireless data transmission, low-cost visualisation, and intuitive interpretation of performance measures.

Some studies have also employed electromyography (EMG) and goniometry to analyse the bowing hand for pedagogical purposes. Martín Armas (2015) found that novice cello students tend to exert excessive tension with the right thumb, a phenomenon that diminishes with increasing years of experience. Her results suggest that elevated muscular activity in the early stages of learning affects the ulnar displacement of the right hand. Accordingly, the author advocates for the systematic analysis of physiological and mechanical factors associated with technical errors in instrumental learning in order to enhance the methodological effectiveness of pedagogical interventions.

The purpose of the present study is to examine the practical applicability of a sensorised glove as an implementation tool in the cello classroom for assisting the learning process of bow control and right-hand technique. The research investigates whether using a glove equipped with force sensors might aid the teaching-learning process in string instrument pedagogy, enhancing or contributing new resources to bowing methodologies. Learning to play a bowed string instrument requires the progressive acquisition of complex sensorimotor skills through the integration of auditory, tactile, proprioceptive, and visual information. From a motor learning perspective, the proposed sensorised glove can be regarded as a wearable system providing concurrent visual augmented feedback in the form of knowledge of performance (KP). Rather than evaluating the musical outcome itself, the proposed system displays the grip forces exerted by the thumb and index finger in real time, allowing performers to monitor a biomechanical variable that is otherwise difficult to quantify during practice. Augmented feedback has been shown to facilitate motor skill acquisition by directing learners' attention towards task-relevant movement variables and promoting self-correction, especially during the early stages of learning; in particular, concurrent visual feedback is considered especially beneficial during the acquisition of complex motor skills, although excessive reliance on continuous feedback may hinder the development of intrinsic error-detection mechanisms (Sigrist et al., 2013).

The study presents a glove with piezoresistive sensors that measures the force exerted by the right hand's index finger and thumb on the cello bow in real time during playing. According to the renowned German cellist and pedagogue Gerhard Mantel (1995, Chapter IX), the index finger is primarily responsible for generating the forces required to sustain and control the bow, as it links the forearm rotation to the bow. Within this pedagogical framework, the middle and ring fingers play only a limited role in applying bow pressure, with the ring finger contributing mainly to stabilising the tilt of the bow. The little finger performs a complementary function to that of the index finger through the opposite forearm rotation, a role that becomes particularly important near the frog, while the thumb is responsible for maintaining the grip of the bow. Based on this pedagogical model, the present study focuses on the thumb and index finger as the principal points of force application during cello bowing.

In the following, Section 2 presents the design and characterisation of the sensorised glove and the methodology used in a small-scale case study with cello students; Section 3 presents the quantitative and qualitative results of the analysis of finger forces and the discussion group with the participants; Sections 4 and 5 finalise with some discussion and directions for future work.

## 2 Materials and methods

The methodology adopted in this study is structured in three main parts: (1) the design and technical development of the sensorised glove, (2) a case study including recordings with cello students, and (3) the evaluation of participants' perceptions of the tool. This approach integrates applied engineering, quantitative analysis of playing technique, and qualitative educational methods to assess the device's technological feasibility and classroom potential.

### 2.1 Technical description: right-hand sensorised glove design and construction

**Figure 1:**
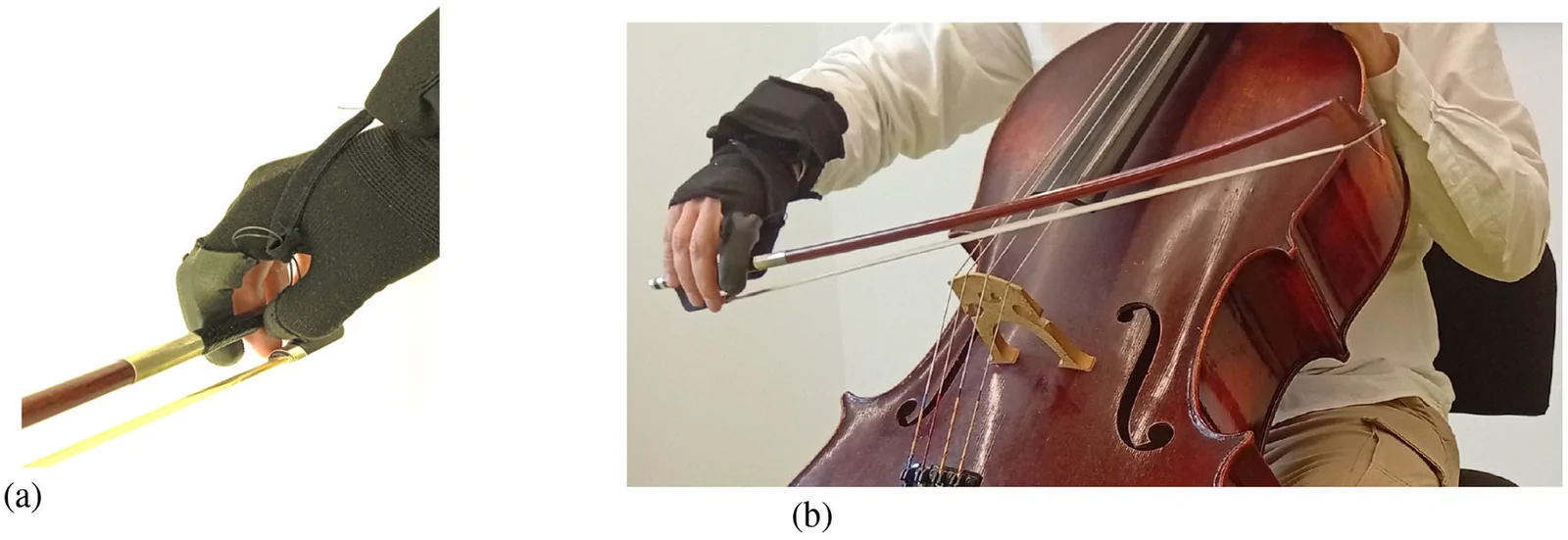
Images of the sensorised glove system. **(a)** Sensorised glove–common bow grip. **(b)** Sensorised glove during performance.

A sensor-equipped glove was designed to capture, in real time, the force exerted by the index finger and thumb on the cello bow (see Figure 1a). Prioritising performer comfort, it features an ergonomic design that tries to minimise interference with playing. Piezoresistive sensors record force data, which are transmitted to a receiving device for visualisation and storage, allowing posterior analysis. The visual graphical interface enables in situ interpretation of the signals, facilitating educational use.

#### 2.1.1 Hardware

For the design of the sensorised glove, a commercially available *Musician's Practice Glove*^2^ made of nylon was selected for its softness, flexibility, and seam-free fabric—qualities that ensure performer comfort and minimal disruption to performance. Non-slip polyurethane caps (Juba PU1400 ECO-PU) were added to the index finger and thumb to preserve a natural bow grip, as polyurethane closely replicates the friction properties of human skin (Derler et al., 2007). The middle, ring, and little fingers of the glove were trimmed for an enhanced feeling of freedom. See Figure 1 for these adaptations. Two piezoresistive force sensors were strategically embedded between the fabric and finger caps —specifically at the finger-bow contact areas of the thumb and index finger—to measure the finger force on the bow in real time. In particular, circular flexible thin-film pressure sensors of the model RP-C18.3-ST were selected, which enable real-time data acquisition, with an operating range covering the physiological range of force of a single finger (see sensor characteristics in Table 1). The RP-C18.3-ST sensor is distinguished by its compact size, flexibility, high stability and consistency. According to manufacturer specifications, the series exhibits a low static temporal drift of less than 5% under a continuous 24.5 N (2.5 kg) load over a 24-h period. While susceptible to minor precision limitations in absolute measurements caused by off-axis forces, they are effective at capturing relative force variations, offering an optimal trade-off for cost-effective and unobtrusive wearable applications.

**Table 1:** Characteristics of RP-C18.3-ST sensors (DFRobot, 2026).

Model	RP-C18.3-ST
Operating range	0.20–58.84 N
Resistance range	< 200 kΩ
Response time	< 10 ms
Sensing area	∅1.45 cm
Thickness	0.4 mm
Static temporal drift	< 5% (24.5 N static load, 24 h)

This choice represents a pragmatic engineering compromise governed by the ergonomic and mechanical constraints of string pedagogy. Alternative sensing technologies, while advantageous in other industrial contexts, present specific limitations for compliant, fabric-based wearable instrumentation. Foil strain gauges—such as those mounted directly onto the wooden bow stick by Guaus et al. (2007)—offer high precision by measuring structural bending; however, integrating them into a flexible textile glove requires rigid backing elements that would immobilise finger joints and alter the natural bow grip. Piezoelectric films generate electrical output only in response to dynamic stress changes, making them inherently unsuitable for tracking quasi-static, continuous finger pressure during sustained bowing. Capacitive force sensors offer high sensitivity but are vulnerable to parasitic capacitance from the human body and signal drift caused by hand perspiration, necessitating multi-layer shielding and moisture encapsulation that increase fingertip stiffness. Thus, despite the aforementioned precision limitations, thin-film FSRs provide the most viable balance of flexibility, mechanical durability, low profile (0.4 mm), and dynamic range for non-intrusive classroom monitoring. To achieve data collection and transmission, the glove includes thin, flexible cables connecting the sensors to a custom-designed printed circuit board (PCB) with a microcontroller. This design guarantees stable signal transmission without compromising the performer's freedom of movement.

The microcontroller employed is an ESP32, a lightweight and energy-efficient platform, where an Arduino environment is used to process and transmit the recorded data wirelessly via Bluetooth to the computer (Windows operating system) for visualisation and recording. While preliminary tests used a wired connection, the implementation of wireless transmission substantially enhanced the musician's ergonomic comfort and mobility. The microcontroller and a 3.2 V rechargeable lithium battery are housed in a custom-made 3D-printed PLA plastic case that protects the electronics and integrates aesthetically with the glove. This case is wrapped in cloth and sewn to the glove's forearm section to ensure unrestricted wrist motion during performance, as shown in Figure 1b.

#### 2.1.2 Signal acquisition and processing

Piezoresistive sensors vary their electrical resistance in response to applied mechanical force. Although factors such as contact area and force distribution introduce inherent variability to the sensor's output, the magnitude of the applied force remains the predominant driving variable, yielding consistent behavioural trends when observed collectively. Furthermore, since the finger–bow contact area remains functionally constant during the task, the interpretation of the results based on relative changes of force magnitude is both clear and reliable.

**Figure 2:**
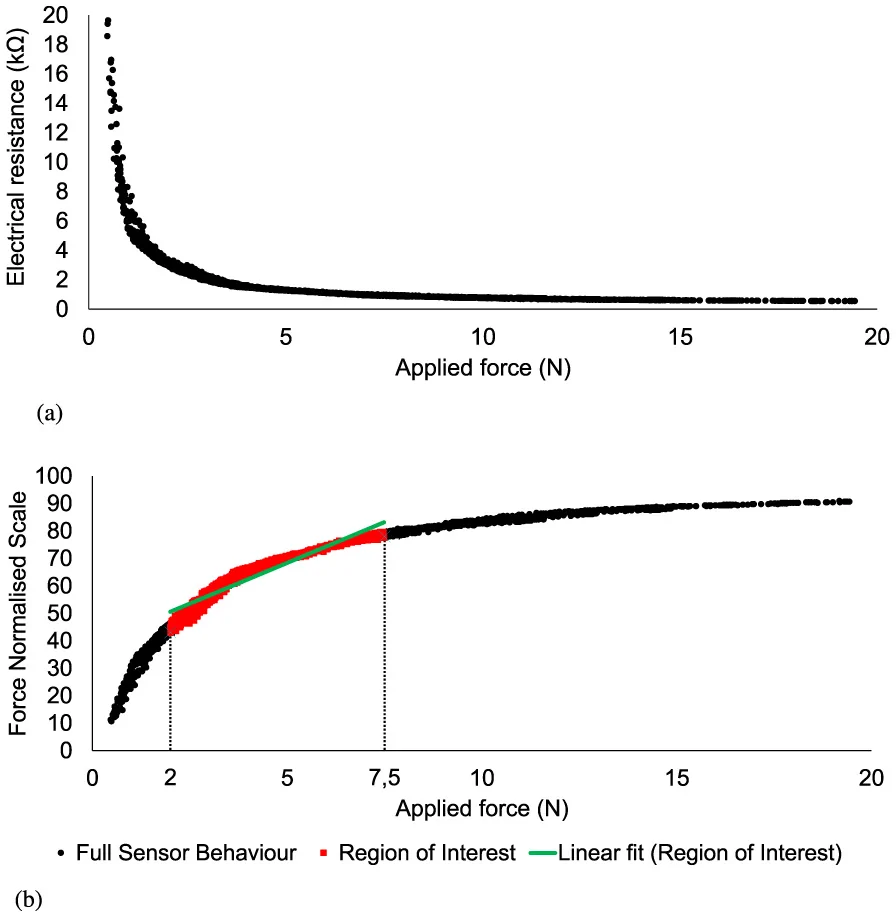
Sensor characterisation: **(a)** electrical resistance reading vs. applied force on the sensor, **(b)** normalised force reading and highlight on the region of interest.

Figure 2a illustrates the sensor characterisation procedure, which was specifically designed to approximate the finger–bow contact in playing conditions. To achieve this, a series of static and centred loads were applied directly by a human finger against the sensor on a calibrated load cell. To characterise the overall electrical response and account for the inherent hysteresis of piezoresistive sensors, the protocol comprised two complete loading and unloading cycles across three distinct sensors, following an initial mechanical pre-conditioning step to stabilise the thin-film polymer. During these cycles, the corresponding electrical resistance values of the sensors were continuously recorded using an Agilent data acquisition system managed via BenchLink Data Logger 3 software (Figure 2a). By ensuring that the finger loads were applied statically and accurately centred during the recordings, off-axis shear forces and viscoelastic drift were effectively minimised. This controlled methodology yielded a highly consistent relationship between the applied mechanical force and the electrical resistance, resulting in lower overall error and hysteresis dispersion than typically expected for flexible FSR sensors in wearable applications.

Since microcontrollers detect voltage rather than resistance, an intermediate conversion stage is necessary. The sensor outputs are therefore processed by a voltage divider circuit that transforms variations in electrical resistance into proportional voltage signals. Consequently, the force exerted by each finger can be expressed as a voltage value. The mathematical relationship governing the operation of the electrical circuit is expressed by the voltage-divider equation as:

$$\begin{array}{l} V_{\text{OUT}}=\frac{R_{1}}{R_{\text{sensor}}+R_{1}}V_{\text{IN}} \end{array}$$ (1)

In the circuit, the input voltage *V*_IN_ supplied by the microcontroller is 3.3 V, whereas the output voltage *V*_OUT_ varies between 0 and 3.3 V depending on the electrical resistance at the sensor *R*_sensor_, which changes with the applied mechanical force (Figure 2a). A high sensor resistance *R*_sensor_ results in *V*_OUT_ approaching 0 V; when *R*_sensor_ is low, *V*_OUT_ tends to 3.3 V. The value of the fixed resistance *R*_1_ is crucial, since it influences both the sensitivity and linearity of the *V*_OUT_ function (Equation 1). For optimal performance, the circuit was designed to prioritise forces between 2 and 7.5 N—the range most relevant for intentional force modulation and refined control in typical bowing, according to preliminary tests on the sensor.

Since numerical force values in newtons or voltage in volts would not be intuitive for performers, the measured *V*_OUT_ is expressed on a normalised 0–100 scale, with 0 and 100 representing the absence and the theoretical maximum of finger force, respectively. Thus, values near 0 correspond to lighter contact forces on the sensor, while those near 100 indicate greater applied pressure. This scale allows the performer to relate the displayed values to the actual intensity of their finger–bow contact and to easily understand variations in the applied force during performance. For completeness, both normalised and absolute finger-pressure values are provided in this study (see Section 3, the Supplementary material and the published database^3^).

To ensure the reliability of this representation, the circuit's response (dependent on the choice of R_1_) was optimised during the design phase to guarantee a stable, linear, and sensitive signal within the range of interest (2–7.5 N). It was also considered that human finger force sensitivity has a just-noticeable-difference threshold of roughly 7%–10% (Pang et al., 1991; Raghu Prasad et al., 2013); therefore, a 7% increase in the applied force was guaranteed to produce a change of at least one unit on the normalised force scale. Regarding linearity, a correlation coefficient of *R*^2^>0.81 was adopted as a reference, a value typically associated with a strong linear input-output relationship (Schober et al., 2021). Figure 2b demonstrates that the established criteria of sensitivity and linearity are met within the range of interest. Consequently, pressure changes perceived by the cellist are clearly and proportionally reflected in the data visualisation, contributing to more intuitive interpretation and effective pedagogical feedback.

#### 2.1.3 Graphical interface and data visualisation

**Figure 3:**
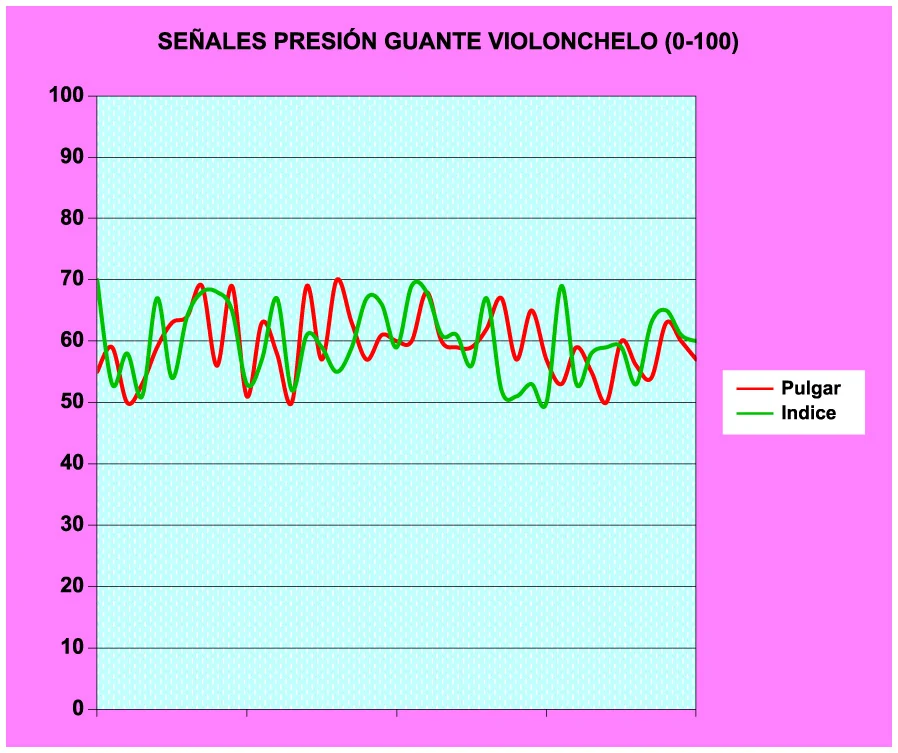
Graphical User Interface (GUI) displayed exactly as seen by the students during the experiment, showing real-time force acquisition during the last 2 s. The red and green signals represent the applied force of the thumb and index finger, respectively. The vertical axis is presented in a normalised scale from 0 to 100. The interface was used in Spanish.

A graphical interface developed in Visual Studio 2022 provides real-time visualisation of the pressure signals from the index finger and thumb, as illustrated in Figure 3. In the graphical interface, the processed signals are displayed in real time as line plots showing the evolution of the pressure applied by each finger during the last 2 s. The *x*-axis represents the samples recorded by the microcontroller (moving time axis), while the *y*-axis shows the processed values within the normalised scale. This graphical format offers the performer a clear visual reference, with the aim of supporting the technical understanding of finger gestures and promoting awareness of the applied pressure at each moment.

### 2.2 Evaluation of the sensorised glove as a tool for the cello classroom

For the validation of the glove as a pedagogical tool, a case study was used as a qualitative methodology. This method has established itself as a suitable approach for studying social, pedagogical, and psychoeducational phenomena, among others. Since it is based on an inductive reasoning process, the researcher engages directly with the reality under study, enabling a deeper understanding of it while fostering creativity and the generation of new knowledge, drawing as well on their own unique personal experience (Angulo Arjona and Noriega Aguilar, 2014). According to these authors, the case study has its roots in the classroom, especially in the management of solved or unsolved cases as didactic and pedagogical tools. In this phase of the study, a cross-sectional, observational and descriptive research design was used (Martín Armas, 2015). It is cross-sectional because there is no continuity over time; observational because data are collected just as they naturally emerge from the sample; and, finally, descriptive because the aim is not to confirm or refute any predetermined hypothesis, but rather to describe the observed phenomena.

#### 2.2.1 Participants and recording procedure

The study sample consisted of five volunteer participants (*N* = 5) who were university-level students specialising in cello performance at the *Conservatorio Superior de Música de Navarra* (CSMN), in the city of Pamplona, Spain. All participants were over 18 years old and had a minimum of 10 years of cello practice experience. Selecting advanced students was a deliberate methodological choice for this exploratory stage: since experienced cellists possess consolidated and healthy motor habits, their recorded profiles provide a preliminary reference of correct execution under concurrent visual feedback, avoiding the erratic grip patterns and excessive tension typical of novices. Prior to data collection—carried out in individual 30-min sessions on 12 April 2024—the sensorised glove was fitted to each participant, followed by a brief instruction on its use, and an explanation of the evaluation instrumentation (glove, hardware, and software). The simplicity of the exercises made it unnecessary to use sheet music or printed material. The rationale for positioning the sensors on the index finger and thumb was also explained. Afterwards, the exercises were described, following a short instrumental warm-up. Once the correct recording of signals (the thumb and the index finger forces) was verified, the designated exercises were carried out. During all tasks, participants continuously viewed the custom-developed graphical user interface (Figure 3) displaying the forces exerted by the thumb and index finger in real time. This visual information allowed them to immediately perceive variations in bow grip force and adjust their technique accordingly, thereby providing real-time visual force feedback. The entire session was recorded in both video and audio. Individual written informed consent was obtained from all participants regarding their voluntary participation and the pseudo-anonymised usage of the collected data.

**Figure 4:**
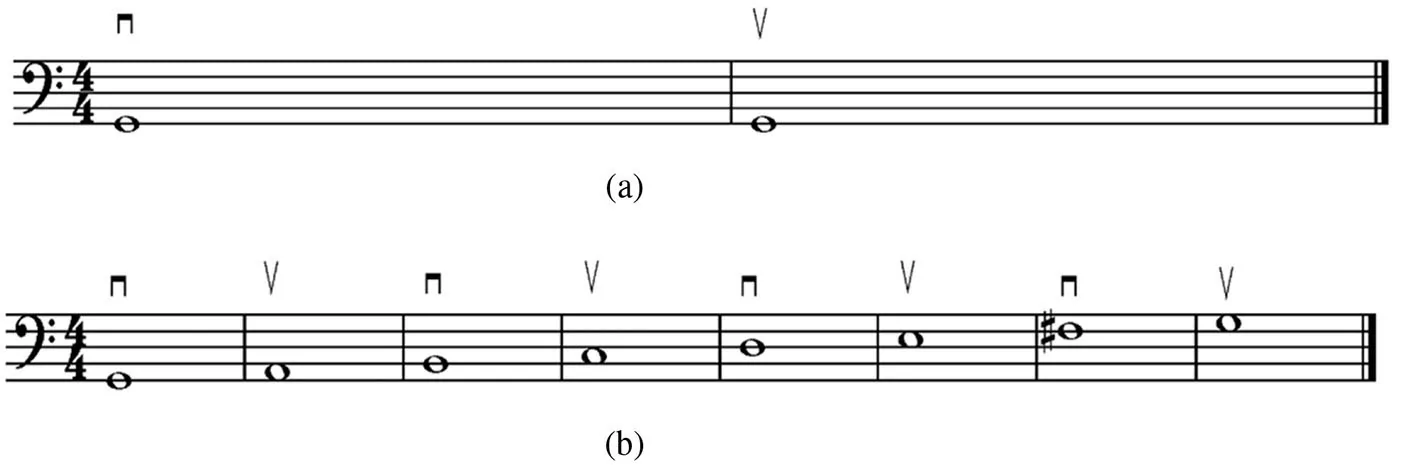
Music score of exercises 2 (one string) and 3 (one-octave G scale). The down-bow (⊓) begins with the bow–string contact closer to the frog and then the contact progresses from the frog towards the tip; whereas the up-bow (∨) starts at the tip and the contact progresses towards the frog. **(a)** Exercise 2. **(b)** Exercise 3.

The students were asked to perform the following three exercises while listening to a metronome clicking. They repeated each exercise at the maximum and minimum possible finger forces. This was done to test the setup's extreme cases. The first exercise was a static one, and the other two regard the *sostenuto* articulation. *Sostenuto*, also called *son filé*, is defined as the production of a sustained, full and stable sound in both volume and bow speed throughout the entire duration of the note, from the frog to the tip, achieved through constant bow contact with the string. Rashkovsky (2023) broadens the concept of *sostenuto*, suggesting that it involves not only sustaining the sound but also the ability to listen to and follow each note attentively from its beginning to its end. The three exercises were recorded as follows:

1. Starting grip: The student holds the bow as usual at the frog, on the G string and at the bow balance point, without moving for four beats (crotchet = 50 bpm, beats per minute). The balance point is the bow position where the bow–string contact happens so that the left and right sides of the bow have the same weight. Repeat for (i) maximum and (ii) minimum possible finger pressure.
2. Sustained note: The student plays two long sustained notes on the open G string (*sostenuto* articulation), as shown in Figure 4a, using the full length of the bow from frog to tip, over four beats (crotchet = 50 bpm) and two bow strokes, starting with a down-bow and maintaining a trajectory equidistant between the fingerboard and the bridge. Repeat for (i) maximum and (ii) minimum possible finger pressure.
3. Musical scale: The student plays a one-octave scale starting on the open G string (G_2_), as shown in Figure 4b, using the full length of the bow from frog to tip, over four beats (crotchet = 50 bpm) with one bow stroke per note, also in *sostenuto* articulation, starting with a down-bow and maintaining a trajectory equidistant between the fingerboard and the bridge. Repeat for (i) maximum and (ii) minimum possible finger pressure.

As a whole, these exploratory tests consisted of five participants, three exercises, two repetitions (maximum/minimum force) and the recording of two finger sensors, leading to a 5 × 3 × 2 × 2 experimental design.

#### 2.2.2 Participants' perception evaluation in a discussion group

Participants were asked questions about their perception and impression of the sensorised glove. To that aim, and based on Krueger's Krueger (1991) framework, the post-intervention discussion group was selected as the methodological tool. According to Krueger (1991), this technique should fulfil five characteristics: bringing people together, being reasonably homogeneous, collecting data, producing qualitative material, and addressing carefully chosen and pre-ordered topics. Moreover, its function may be to identify trends and shed light on the perception of a particular product, defining this practice as a structured conversation conducted within a permissive, non-directive environment, in which participants influence one another through their responses to the ideas and comments that arise during the discussion (Krueger, 1991). In this regard, the question guide—open-ended, non-dichotomous, reflective, and well-formulated (Krueger, 1991)—was validated by two cello professors and structured into four thematic blocks:

- Ergonomics and comfort
- Ease of interpreting the graphical interface
- Use as a tool for self-monitoring within a pedagogical process
- Awareness of the pressure exerted while playing

The sample consisted of the same five cello students that had previously participated in the recordings (5 days before). It was expected that, although homogeneous, participants' profiles would also present heterogeneous opinions to enable debate and contrast of ideas. The moderator was the researcher themself (first author), guiding the interventions and fostering an atmosphere of spontaneity and relaxation.

The discussion group took place on 17 April 2024 and it was conducted in Spanish, the common language of cello instruction in the host institution CSMN. As Krueger (1991) recommends, a brief introductory conversation was held before the session to reassure participants and create a warm, friendly environment. The five study participants were invited at 9:00 a.m. to a room at the CSMN, where six chairs were arranged in a closed circle. The methodology of the discussion group was explained, with some instructions regarding verbal participation (respecting turns, completing statements, and feeling free to express themselves spontaneously). The session lasted 60 min and was structured around the four items mentioned above. Audio from the session was recorded—with the explicit consent of all participants—for subsequent analysis of the interventions.

### 2.3 Data processing and preparation

**Figure 5:**
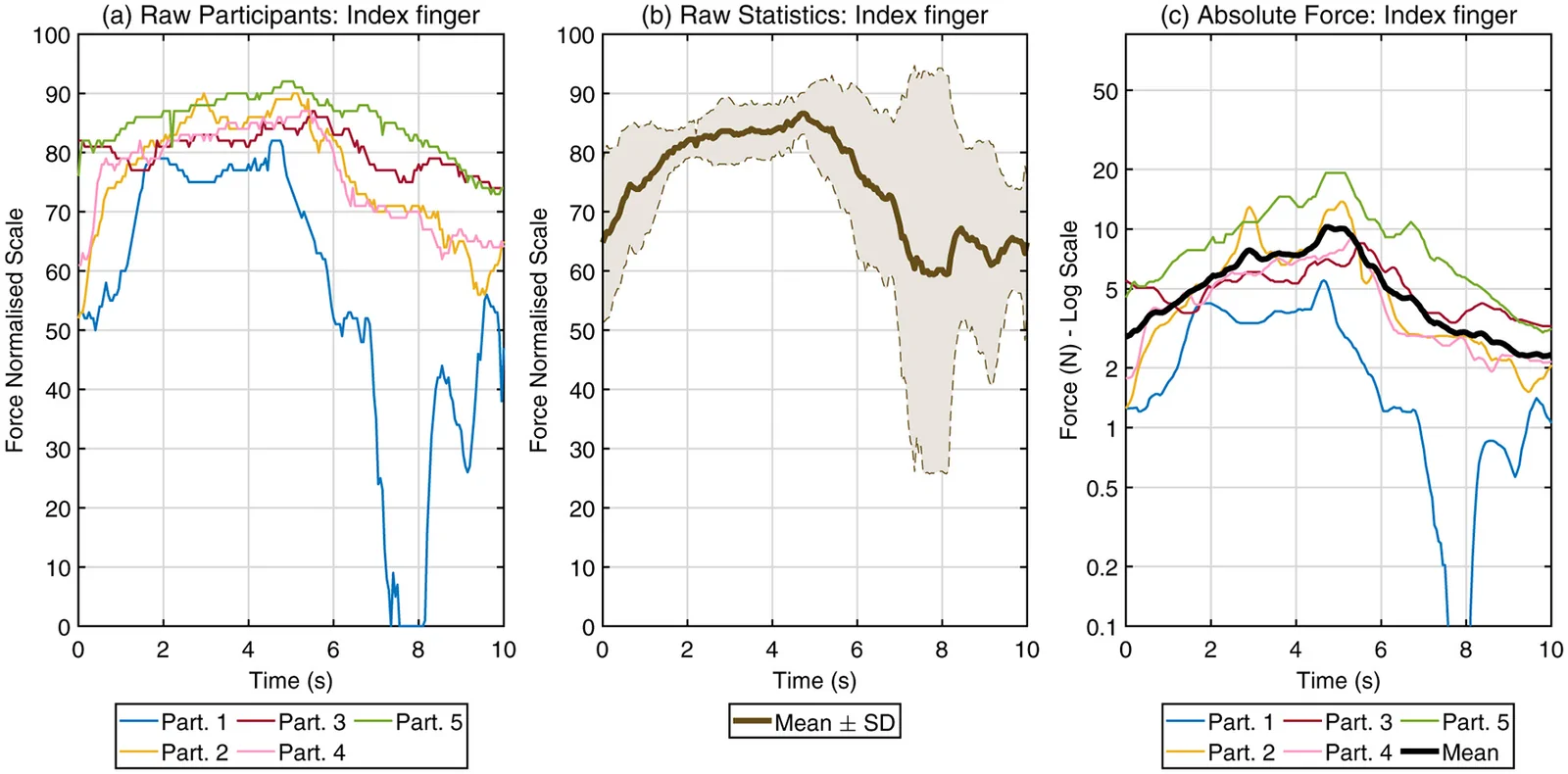
Three representations of the recorded data: **(a)** raw data per participant in the normalised scale as they observed during playing, **(b)** averaged data considering all participants and a grey area indicating the standard deviation in the normalised scale, **(c)** force measurements given in a logarithmic scale with overlapping mean signal across all participants. Data for exercise 2, index finger, maximum force.

**Figure 6:**
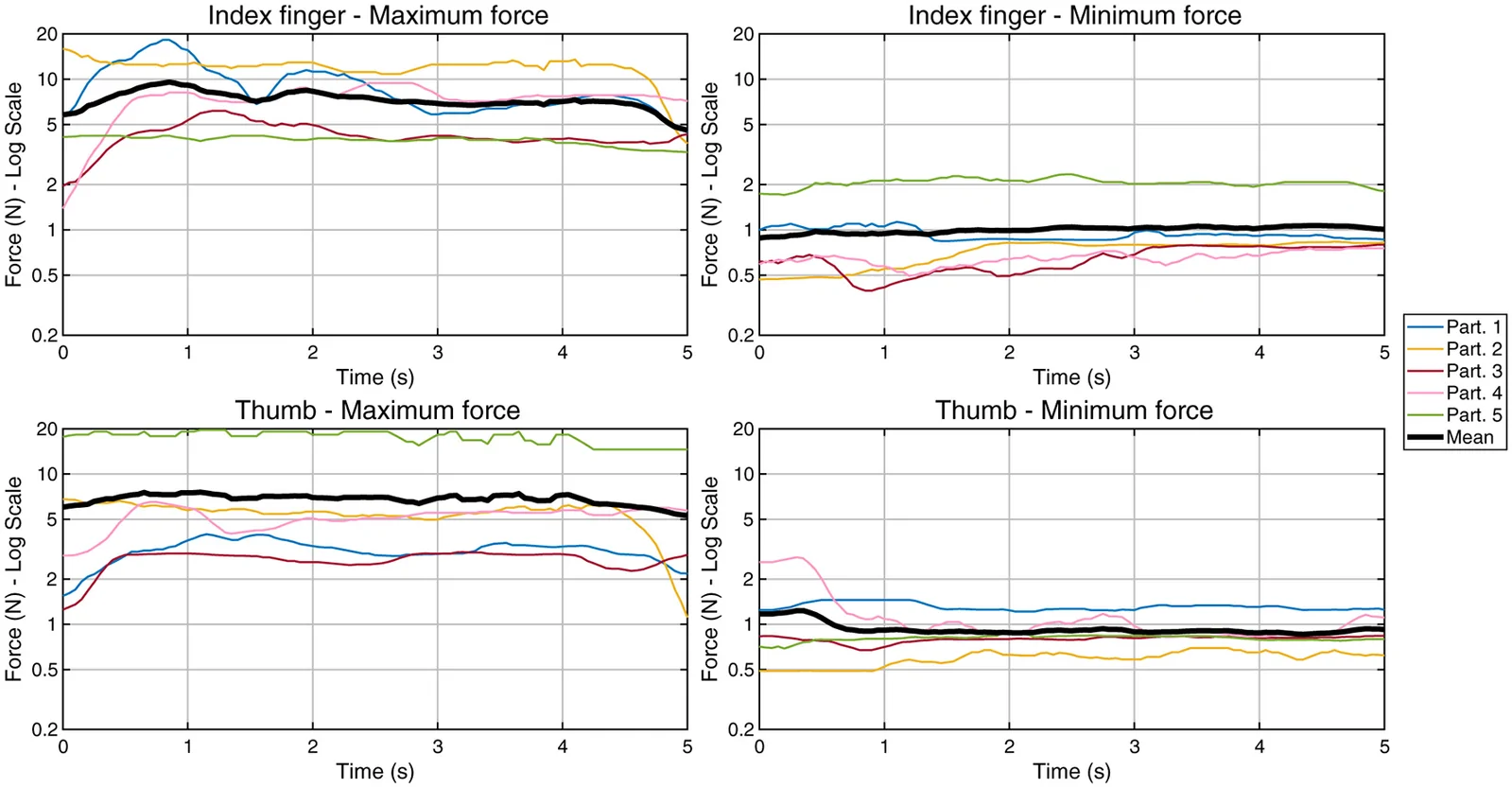
Force measurements by all players (in thin coloured lines) and their mean (bold black line) for the first exercise, consisting of holding the bow on the G string for about 5 s.

**Figure 7:**
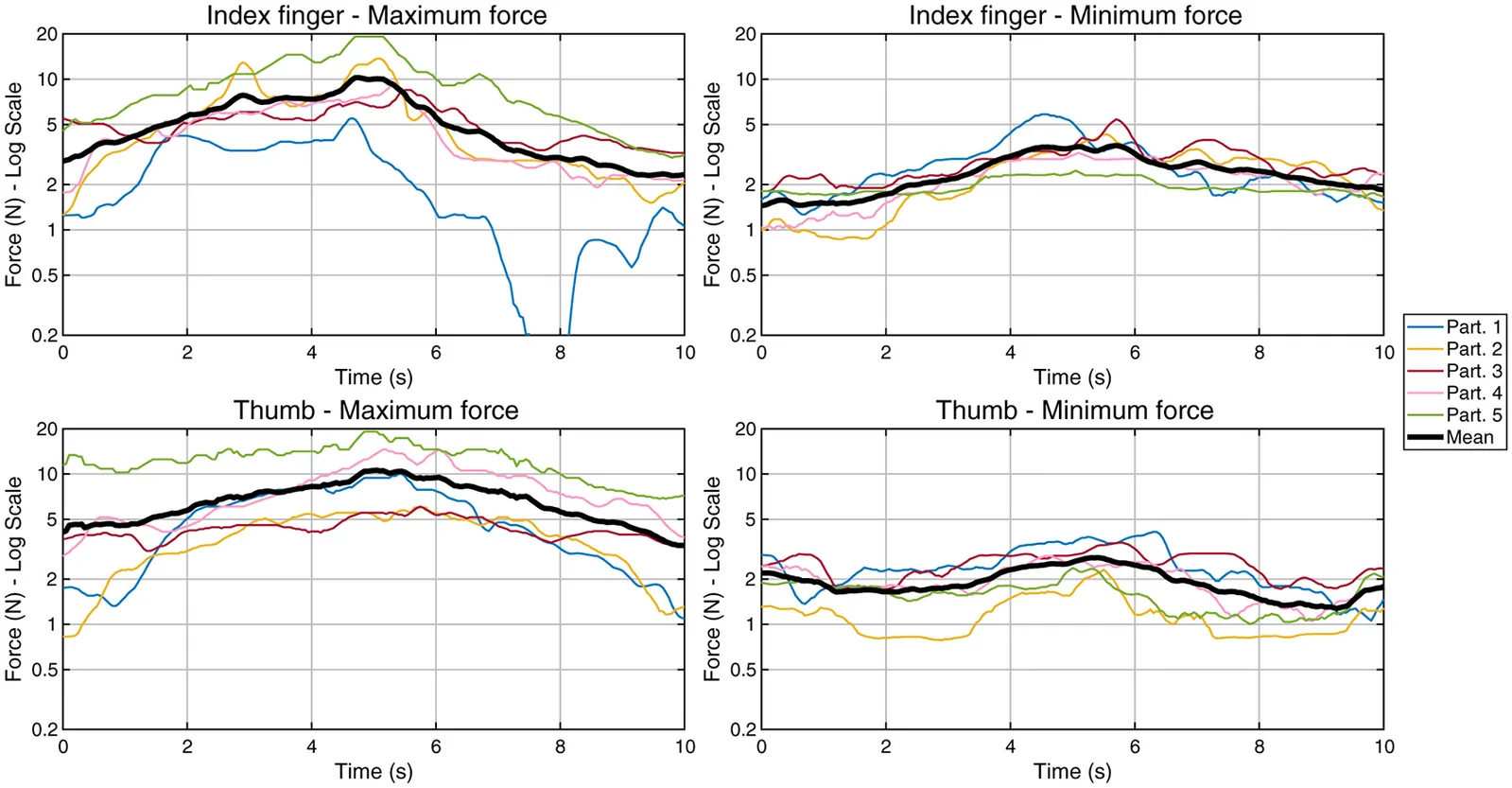
Force measurements by all players (in colour) and their mean (black) for exercise 2, consisting of playing two whole bows on the G string, starting down-bow (moving from the frog towards the tip) and then up-bow (from the tip to the frog); each note took four pulsations at 50 bpm (Figure 4a).

**Figure 8:**
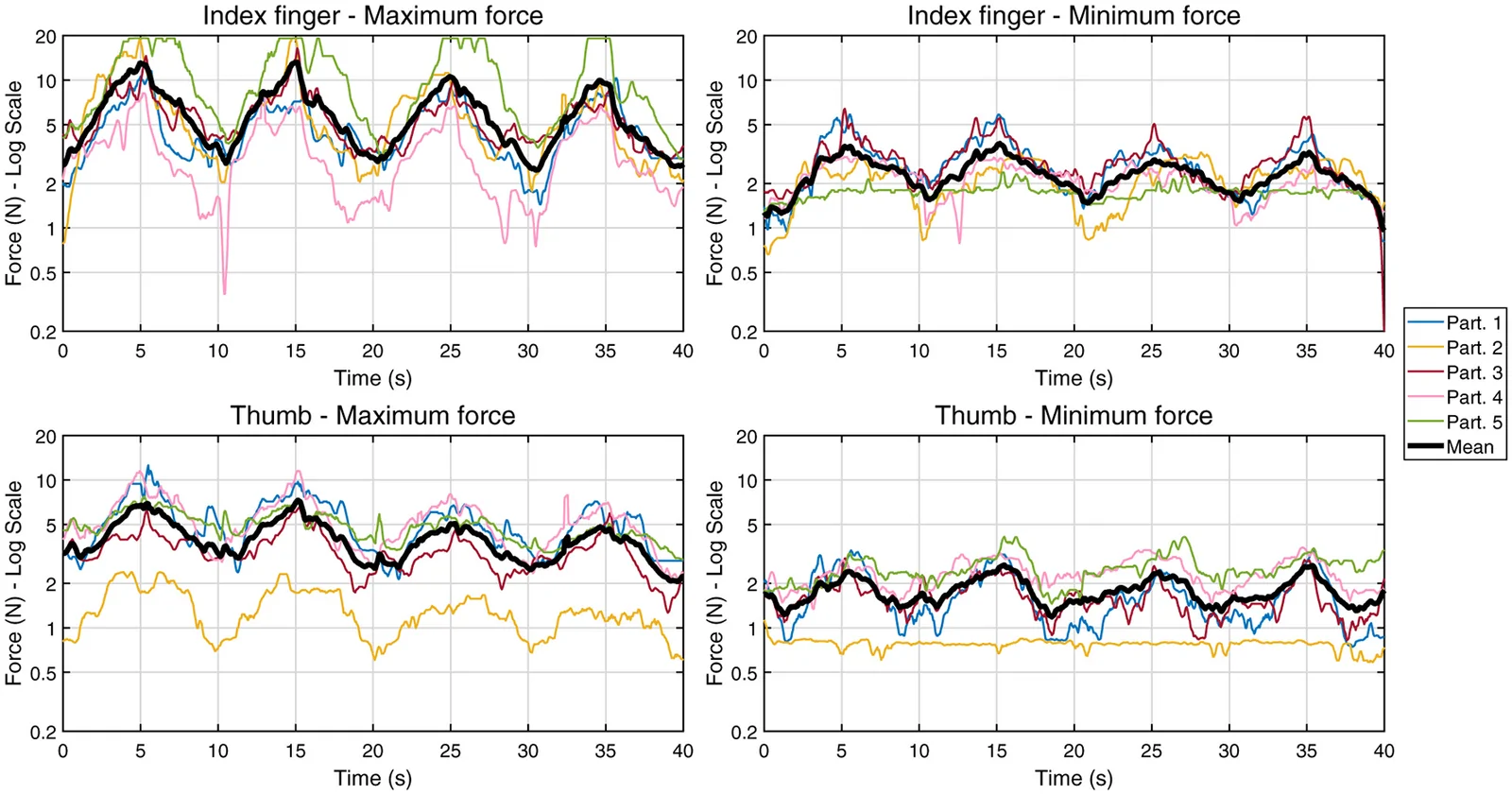
Force measurements by all players (in colour) and their mean (black) for the third exercise, consisting of playing a one-octave G scale starting down-bow at the open G string; each note took four beats at 50 bpm (Figure 4b).

The raw force signals in the normalised scale were sampled at 20 Hz and exported in CSV format (including the time signal) for subsequent offline analysis. The data can be visualised in the normalised scale for each participant (see an example for exercise 2 in Figure 5a), as well as in the averaged normalised-scale values considering all participants (Figure 5b). These two forms are the two most straightforward possibilities that can be easily obtained using available spreadsheet software. Therefore, these might be the two visualisations most relevant for pedagogical purposes. To allow comparison with related performance-science studies, though, the finger force data will be shown in Newtons in this publication (Figures 5c–8). The Supplementary material includes analogous plots to Figure 5 for the recorded exercises. Data processing and visualisation for this publication was performed using MATLAB (by MathWorks).

To convert the values of the normalised scale (0–100) into absolute force (N) across the entire recorded spectrum, a previously characterised calibration curve mapping forces up to 20 N was used (see Figure 2b). Due to the inherent non-linear electrical response of the force-sensing resistors (FSR), the conversion was performed using piecewise cubic Hermite interpolation. This numerical method was applied across the full operational range, ensuring a smooth and monotonic transformation of the raw data into Newtons while preserving the intrinsic response behaviour of the sensor.

Subsequently, as commonly done for slow-varying signals—as in recorded human motion—, a filter was applied to smooth high-frequency fluctuations caused by electronic noise, minor mechanical vibrations, and signal quantisation that were not directly related to the applied finger force. To do that, the force values (Newtons) were processed using a moving average filter with a centred window of five samples (corresponding to 0.25 s at 20 Hz). The five-sample moving-average filter had a (–3)-dB frequency of approximately 1.8 Hz and its first spectral zero at 4 Hz. It therefore largely preserved variations at approximately 1 Hz and below while progressively attenuating faster fluctuations (see Figures 5a vs. 5c). Finally, the processed data were represented on a logarithmic scale for visualisation purposes. This representation allows force values in Newtons to be displayed while preserving the perceptual character of the recording, since human perception of intensity is usually expressed in the logarithmic scale. A direct comparison between linear and logarithmic force scaling for a representative bowing task is provided in Section 1.2 of the Supplementary material. Furthermore, as a result of using the logarithmic scale, the visualisation of the data exhibits curves that are similar to those of the original normalised scale, thereby facilitating interpretation of the signals (Figure 2b).

Piezoresistive force-sensing resistors (FSR) are known to exhibit uncertainties in absolute force measurements due to their sensitivity to contact area and force distribution (Lebossé et al., 2011). This study addresses these limitations through two complementary strategies. Mechanically, the finger-bow contact area was kept functionally constant during tasks, with polyurethane caps helping to homogenise pressure distribution over the sensing area. Methodologically, absolute force values were analysed statistically using the aggregated mean and standard deviation (*Mean*±*SD*) across the five cellists. Data from each participant are given in Figures 6–8 for visualisation purposes, yet the mean values (in bold) are to be considered. From a statistical perspective, computing the mean across *N* = 5 independent executions effectively mitigates the sensors' inherent random noise. According to the Standard Error of the Mean (SEM) principle ($SEM=\sigma /\sqrt{n}$), this aggregation mathematically reduces the random measurement error by approximately 55% ($\approx 1/\sqrt{5}$) compared to a single observation. Furthermore, rather than relying on individual measurements—which are particularly prone to idiosyncratic sensor placement or specific finger anatomies—this multi-subject approach helps mitigate individual biomechanical peculiarities. Consequently, the resulting aggregated data yields preliminary baseline reference ranges for each bowing technique within this specific group of advanced cellists.

## 3 Results

### 3.1 Finger–bow force analysis

In Exercise 1, which consisted of holding the bow immobile on the G string, the aggregated mean values (black bold lines in Figure 6) show that the maximum finger–bow force stabilised at 7.33 ± 3.72 N for the index finger and 6.80 ± 6.18 N for the thumb (see Table 2 for the mean values of all recorded exercises). When the same exercise is performed in a relaxed position, the average minimum finger–bow force to maintain the bow in balance on the string without moving was 1.00 ± 0.61 N for the index finger and 0.93 ± 0.33 N for the thumb. Considering the cellists individually, one can observe that, at the maximum-force recordings, some participants begin at a lower contact pressure (P1, P3, P4) while some begin directly at the maximum pressure (P2, P5). This difference is explained because the first group began the exercise with a force sufficient to hold the bow and increased to maximum during the first 0.5 s, while the other group was already holding at maximum before the recording started. We see the opposite effect in the minimum-force plots for players P3 (index) and P4 (thumb), who tried to reduce the force during the first second of the execution. It is also worth mentioning that participant 5 (in green) tends to perform at extreme high pressure (see thumb-maximum and index-minimum plots), a pattern that repeats in exercises 2 and 3.

**Table 2:** Mean force and standard deviation (SD) in Newtons comparing exercises and fingers.

Exercise	Index max	Thumb max	Index min	Thumb min
	Mean ±SD (N)	**Mean** ±**SD (N)**	**Mean** ±**SD (N)**	**Mean** ±**SD (N)**
1	7.33 ± 3.72	6.80 ± 6.18	1.00 ± 0.61	0.93 ± 0.33
2	5.19 ± 2.69	6.69 ± 3.78	2.40 ± 0.60	1.92 ± 0.64
3	6.03 ± 3.08	4.01 ± 1.84	2.30 ± 0.62	1.78 ± 0.77

While data conversion via Hermite interpolation covers the full spectrum up to 20 N, force readings exceeding the upper boundary of the Region of Interest (7.5 N) exhibit reduced sensor sensitivity and should be interpreted with caution regarding absolute precision.

Exercise 2 consisted of playing a sustained note for two full bows on the G string (*sostenuto* articulation, four beats for the down-bow and four beats for the up-bow, at 50 bpm; Figure 4a). The average values for the index finger (black bold lines in the top plots of Figure 7) show that both the maximum and the minimum cases show an increasing trend whose peak occurs when the tip of the bow reaches the string (time *t* = 4.8 s), descending again as the bow–string contact returns to the frog. This behaviour corresponds to the nature of the *sostenuto* articulation, where the player tries to maintain a constant pressure between the bow hair and the string. Since the bowing hand is placed at one extreme of the bow, less pressure has to be exerted when the lever is shorter (at the frog) than when the lever is longer (at the tip). When performed with maximum intentional pressure, the aggregated mean force ranged from a minimum of 2.29 ± 0.82 N at the frog to a peak of 10.26 ± 5.69 N at the tip (*t* = 4.8 s). Conversely, when executed with a relaxed grip, the values fluctuated in a much narrower physiological band, ranging from a minimum of 1.45 ± 0.40 N to a peak of 3.63 ± 1.16 N (*t* = 5.7 s). When considering participants individually, the first striking aspect is the behaviour of P1 (in blue in Figure 7) in the index-maximum plot: P1 exhibits a drop in the measured index-finger force in the interval between 7 and 8 s. This could be explained by a complete release of the finger at this interval, or as a result of curling the index finger around the bowstick in a so-called hook shape, in which the sensor might lose contact with the bowstick.

The results for the thumb (lower part of Figure 7) reveal similar behaviour to those of the index finger during the maximum-force recording, characterised by increasing force up to the bow change (peaking at 10.60 ± 5.93 N) followed by a decrease until the bow frog is close to the string again (dropping to 3.34 ± 2.45 N)—due to the force required depending on the lever length to maintain the bow force on the string. Yet a worth-noticing difference appears when the fingers are relaxed (minimum force plot). Here, at the thumb signal, a similar W-trend appears for all players: the aggregated mean thumb force reaches a local minimum when the bow is at equilibrium, increases until reaching its maximum of 2.78 ± 0.68 N when the bow tip is on the strings (*t* = 5.4 s) and then analogously reaches a minimum again of 1.28 ± 0.32 N before the end of the execution. On the one hand, these two local minima correspond to the situation where the bow is at the balance point, i.e., on the string with the left and right sides of the bow in equilibrium. On the other hand, the increased thumb force at the beginning and end of the exercise (when the bow–string contact happens close to the frog) might be required to support the bow on the string, that would otherwise tremble and turn around its axis. As explained for the other three plots, the increase in force at the tip (*t* = 5 s) is also due to the need to apply greater force with the fingers to counteract the lever effect in that position.

Exercise 3 involved playing a one-octave scale starting at the open G string (Figure 4b), alternating full bow motions starting with down-bow (from frog to tip). Considering the maximum force recordings, the average values for both the index finger and thumb (black bold lines in the top plots of Figure 8) show similar behaviour, with higher peak values for the index finger (13.21 ± 6.02 N) than for the thumb (8.69 ± 2.35 N). As observed in the maximum-force measurement of exercise 2, the force that the fingers exert on the bow is minimal when the bow rests on the string close to the frog (2.46 ± 1.29 N for the index and 2.42 ± 0.60 N for the thumb) and is maximal when the bow-string contact happens close to the tip, that is, at the position of the bow change from down-bow to up-bow. In the index-maximum-force plot, participant 4 (in pink) exhibits some irregular peaks in force, including an index-finger momentary release at around *t*≈10 s. Participant 5 shows the highest forces, with values above 20 N, that have been cropped at 20 N for visualisation and for being unrealistic during playing.

Regarding the relaxed situation (minimum force, Figure 8), differences appear between the index finger and the thumb. Like in exercise 2 (see Table 3), the index finger follows an increase-decrease pattern in force that corresponds one-to-one with the motion of the bow on the string: the index-finger force increases during the down-bow (peaking at 3.72 ± 1.80 N) and decreases during the up-bow motion (down to 1.55 ± 0.42 N). The thumb signal has a slightly smaller dynamic range, with peak values of 3.13 ± 0.74 N and minimums of 1.28 ± 0.53 N. Here, if every bow stroke is considered individually, the thumb signal follows the W-pattern described for exercise 2. One observation must be made for player 2 (in orange), which showed much lower thumb values both for the maximum and the minimum force tasks. Since no clear indices appeared that the equipment was malfunctioning, one must interpret this as an off-axis measurement, where the player did not press at the centre of the FSR sensor.

**Table 3:** Range of execution (maximum and minimum values and their standard deviation in Newtons) and an indication of the position of the bow at each given value; comparing exercises 2 and 3 for both fingers.

Exercise	Index finger		Thumb	
	**Min** ±**SD (N)**	**Max** ±**SD (N)**	**Min** ±**SD (N)**	**Max** ±**SD (N)**
Ex. 2 max. force	2.29 ± 0.82	10.26 ± 5.69	3.34 ± 2.45	10.60 ± 5.93
	Frog	Tip	Frog	Tip
Ex. 2 min. force	1.45 ± 0.40	3.63 ± 1.16	1.28 ± 0.32	2.78 ± 0.68
	Frog	Tip	Balance point	Tip
Ex. 3 max. force	2.46 ± 1.29	13.21 ± 6.02	2.42 ± 0.60	8.69 ± 2.35
	Frog	Tip	Frog	Tip
Ex. 3 min. force	1.55 ± 0.42	3.72 ± 1.80	1.28 ± 0.53	3.13 ± 0.74
	Frog	Tip	Balance point	Tip

Values exceeding 7.5 N fall outside the linear Region of Interest (ROI); due to piezoresistive compression, peak absolute force measurements above this threshold present reduced resolution compared to readings within the 2.0–7.5 N ROI.

### 3.2 Discussion group

The opinions expressed in the focus group are considered to evaluate the usage of the sensorised glove in a teaching situation (see summary in the Supplementary material); and analysed in the four thematic blocks presented in Section 2.2.2. Concerning their physical sensations while playing with the sensorised glove and assessing its ergonomics and comfort: Participant P4 appreciated that the glove leaves the middle, ring, and little fingers free but expressed some concern that the sensors might move during playing. P1 found the glove comfortable but noted that it altered the tactile “real” sensation and limited hand mobility; P5 expressed similar views. P2 considered the sensors too rigid and large, whereas P3 found the glove comfortable and expressed that the performance of the exercises was unaffected by sensor rigidity. Regarding the ease of interpreting the graphical interface, P2 and P5 described it as intuitive, highlighting the use of separate colour-coded lines for the index finger and thumb. P1, P3, and P4 suggested including a “normal use” range accompanied by alerts for over- and under-pressure zones. As for the glove's usefulness as a pedagogical tool, P2 regarded it as highly promising beyond bowing exercises, particularly for self-monitoring during personal practice. P5 considered it helpful for raising awareness of applied pressure and for preventing injuries. P4 felt that the “non-real” sensation of playing with the glove might reduce its pedagogical effectiveness. P3 pointed out that a sensor-equipped glove helps to objectify the subjective sensation of “pressing”. The moderator emphasised the importance of keeping the glove low-cost to enable its potential use in cello classrooms. Finally, concerning awareness of applied force while playing, P4 observed that during bow changes at the frog, contact and pressure on the index finger tended to decrease, and the glove helped to raise awareness of this phenomenon. P1 realised that increased pressure with the index finger led to a hooked finger shape and potentially a loss of weight. P2 reported that the glove enhanced awareness of *sostenuto*, allowing them to perceive the gradual decrease in weight while playing. P3 noticed that pressure increased towards the tip to compensate for the natural loss of weight at that point.

For cellists, “loss of weight” refers to the diminishing capacity to transfer the arm's own weight to the bow as it moves towards the tip. At the frog, the arm is closer to the body, allowing its weight to be transmitted easily to the bow, aided by the bow's leverage–no extra pressure is needed to sustain the sound. As the bow approaches the tip, the arm extends and the transmission of its weight becomes less efficient; since the bow is further from the body's centre of gravity, contact with the string weakens and must be compensated.

## 4 Discussion

The present findings suggest the technical feasibility and perceived pedagogical value of the sensorised glove. By integrating flexible instrumentation into an authentic teaching environment, the study demonstrates its technical viability as a low-cost wearable providing real-time visual feedback in the cello classroom. The glove was able to track finger force variations during bowing, suggesting a functional balance between monitoring capability and limited physical interference. Moreover, the discussion group highlighted notable learning benefits, particularly in fostering awareness of bow-hand control during self-monitoring, while also expressing ergonomic concerns related to the rigidity of the sensors.

From a quantitative perspective, aggregated force data empirically indicate the appropriateness of the selected Region of Interest (ROI) between 2.0 and 7.5 N (Table 2). During dynamic bowing (Exercises 2 and 3), the minimum grip force required for stable bow control converged around 2.0 N (e.g., a mean of 1.92 ± 0.64 N for the thumb), whereas the maximum mean force exerted across all tasks reached 7.33 ± 3.72 N (Exercise 1, index finger). The chosen ROI effectively represents the averaged ranges observed in the recordings (Table 2), capturing key physiological thresholds for pedagogical assessment while minimising distortion from isolated force peaks. Regarding the possibility of off-axis contact, stable sensor contact was observed in nearly all measurements—only one clear off-axis contact was observed (thumb; Figure 8). Finger caps helped preserve alignment, and across the 60 dynamic measurements presented, the relative force patterns remain pedagogically valid.

In addition to spatial stability, temporal stability is essential for reliable force monitoring. Although piezoresistive sensors can exhibit viscoelastic drift under continuous static loading, such degradation was negligible within our experimental timeline. While student sessions lasted approximately 30 min, physical data acquisition occurred during brief, discrete exercises (5–40 s) separated by frequent recovery pauses that allowed the polymer substrate to recover elastically. Combined with the low inherent viscoelastic drift of the RP-C18.3 series (see sensor characteristics in Table 1), the system exhibited sufficient temporal stability for the duration of these short exercises. Nevertheless, no repeat calibration was performed within or across recording sessions, and consequently in-session sensor sensitivity drift was not quantified. Furthermore, the system architecture has been conceived to accommodate automated baseline zeroing (taring) during natural playing pauses in future studies, thereby resetting the zero-load reference and compensating for long-term polymer viscoelasticity.

Having established the spatial and temporal stability of the system, the pedagogical validity of these dynamic force patterns lies in their capacity to translate abstract biomechanical principles into actionable, real-time visual feedback. Relying solely on tactile proprioception during playing can be misleading, as musicians often struggle to accurately gauge how grip pressure changes across the bow length. As participant P3 noted, the system “helps to objectify the subjective sensation of ‘pressing”'. A clear classroom example is the *sostenuto* articulation (Exercises 2 and 3). When drawing the bow from frog to tip, the arm extends away from the body's centre of gravity, resulting in a natural loss of arm weight. As recorded under concurrent visual feedback, the force trajectories indicate that maintaining an even tone involves increasing index-finger pressure towards the tip to compensate for the lengthening lever and reducing it again towards the frog. Visualising this dynamic force pattern on the graphical interface enables students to verify whether their force modulation follows the pattern observed across the exercises. Furthermore, the live display helps expose unconscious habits, such as participant P4 recognising an unintended drop in index-finger contact during bow reversals at the frog—a subtle error that tactile sensation alone had failed to flag. Concerning the assessment of bowing positions, the relationship between grip-force patterns and specific bow positions was inferred from the standardised timing of the experimental protocol rather than directly measured. Further studies might incorporate synchronised video or motion capture recordings to validate these temporal correspondences.

These outcomes suggest that the glove effectively captures bowing behaviour within the physiological range of healthy technique, confirming its reliability for monitoring intra-subject motion patterns and identifying biomechanical principles underlying sound production without severely disrupting performance. As evidenced by the focus-group feedback, while wearable instrumentation inevitably introduces slight alterations to tactile feedback (as noted by P1 and P5), trimming the glove effectively preserved the freedom of the non-gripping fingers (P4), and sensor rigidity did not impede the physical execution of the bowing exercises (P3). Yet certain technical and methodological limitations should nonetheless be acknowledged. The proposed system is based on force measurements restricted to the thumb and index finger. Although these digits are considered to play a central role in bow grip according to established pedagogical approaches, effective bow control results from the coordinated action of all fingers. The middle, ring, and little fingers also contribute to balance, stabilisation, and fine motor adjustments throughout the bow stroke. Therefore, the proposed glove should be interpreted as capturing key aspects of bow grip force rather than providing a complete biomechanical description of bow control. Furthermore, from an electronic design perspective, the signal acquisition system relies on a voltage divider circuit, a design choice intended to minimise the component count and streamline the wearable's hardware architecture. While this configuration provides sufficient pseudo-linearity across the core pedagogical range (2.0–7.5 N), resolution decreases at higher forces as the piezoresistive material nears saturation, flattening its resistance response. Consequently, data dispersion at elevated pressures likely reflects both complex contact stresses and reduced sensor sensitivity. Nevertheless, because such extreme pressures are pedagogically undesirable and fall well outside the bounds of healthy bowing technique, this upper-range limitation does not compromise the tool's utility.

All force data reported in this study were recorded while participants were watching the real-time visual display of their own finger forces (Section 2.1.3). The recorded force trajectories should therefore be understood as bowing behaviour under concurrent visual feedback. It is also possible that, with a 0–100 scale on the display, some participants may have understood the instruction to play at "maximum finger force" as pushing the displayed number towards its ceiling, rather than reaching a true physiological maximum. The values recorded for Participant 5, which exceeded 20 N and were already described as unrealistic for playing, would be consistent with this possibility, even though this cannot be confirmed from the present data.

While the small cohort of five advanced participants (*N* = 5) limits the immediate generalisation of the absolute values, their profiles provided an initial reference framework. From a pedagogical perspective, the intended end-users of the sensorised glove span from beginners who stand to benefit most from real-time visual feedback to avoid excessive thumb tension and understand bow lever mechanics to intermediate and advanced players seeking to self-monitor and fine-tune complex articulations during individual practice. To mitigate the sample limitations, our analysis prioritised dynamic relative force changes and aggregated data (*Mean*±*SD*) rather than absolute thresholds.

Although participation was voluntary and students were informed that their responses would remain confidential and would not influence their academic evaluation, the dual role of the first author as both cello teacher and moderator of the discussion group may have introduced social desirability bias, potentially reducing participants' willingness to express critical opinions. Future studies could consider using an independent moderator to minimise this potential source of bias.

Overall, the sensorised glove demonstrates potential as a pedagogical aid in string-instrument instruction. By enabling students to visualise and self-monitor pressure during bowing, it facilitates greater autonomy, awareness, and control in developing right-hand technique. The visualisation method provides clear and intuitive feedback during performance: signal smoothing and logarithmic scaling help align the displayed force values with students' perceptual experience, making it easier to relate the shown graphics to bow–hold sensations.

## 5 Conclusions

The results show that the glove-based system reliably captures the characteristic bow-hand force patterns underlying fundamental cello bowing techniques, revealing how players modulate index-finger and thumb pressure in response to lever-length effects and bow articulation demands. In both sustained notes and scale playing, the measured forces follow the expected biomechanical logic of the bow as a lever, with higher finger pressures at the tip and reduced forces near the frog. There is a trend that the index finger exerts higher forces than the thumb, with the thumb presenting minimum values at the balance point of the bow on the string. The data also revealed a distinct difference between holding and moving the bow: initiating motion, even in a relaxed manner, activates higher finger pressure than maintaining a static hold. Although the increase in finger force towards the bow tip can be explained by the changing mechanical leverage of the bow, it is also consistent with principles of sensorimotor control. Rather than simply reacting to mechanical constraints, performers continuously adjust grip forces to regulate the interaction between the bow and the string, thereby aiming at a stable sound quality throughout the bow stroke.

The focus group feedback highlighted the advantages and limitations of the sensorised glove. Using the sensorised glove enhanced players' awareness of *sostenuto*, illustrating that, as the bow moves towards the tip, they experience a loss of weight of the arm, which they must compensate for with increased finger pressure. Participants generally appreciated the ergonomic design of the glove, the intuitive graphical interface, and the possibility to visualise pressure distribution over time, thereby promoting awareness of bow force and right-hand technique. Yet the glove's rigidity and altered tactile sensation were perceived as the main factors limiting its naturalness and comfort. Future research should build upon the presented pedagogical analysis and extend it by including longitudinal pedagogical interventions. Within an A/r/tographic framework, one could explore its integration into cello lessons over time. Additionally, combining the glove with synchronous audio recordings would allow detailed correlation between applied forces and resulting sound intensity, enriching understanding of the biomechanical–acoustic relationship in performance.

The findings suggest that a low-cost sensorised glove represents a promising pedagogical tool for string instrument instruction, since it can make finger forces visible and quantifiable. It can support focused exercises related to bowing technique and sound production, encourage self-monitoring during individual practice, and increase the awareness of applied pressure. Beyond its role as a learning aid, the glove may also have potential to support ergonomic awareness during practice, although its effectiveness in injury prevention remains to be investigated.

## Data Availability

The datasets presented in this study can be found in online repositories. The names of the repository/repositories and accession number(s) can be found below: https://doi.org/10.5281/zenodo.19551529.

## Footnotes

1 https://www.keithmcmillen.com/labs/k-bow/, accessed March 2026.

2 https://musicianslive.org/, accessed on March 2026.

3
https://doi.org/10.5281/zenodo.19551529
