# Supplementary material for: Force-sensing glove with real-time visual feedback for cello right-hand technique instruction
Source: Front Psychol. 2026 Sep 3;17:1856906. doi: 10.3389/fpsyg.2026.1856906 (PMC13582492; doi:10.3389/fpsyg.2026.1856906)
Supplement: Supplementary file 1 [file Data_Sheet_1.pdf]

Supplementary Material

1 SUPPLEMENTARY FIGURES AND TABLES

This supplementary material provides additional data supporting the findings of the main study. It is divided into three sections: Section 1.1 presents the complete set of graphical representations for the quantitative finger-bow force data, Section 1.2 provides a comparative representation of linear versus logarithmic force scaling, and Section 1.3 details the qualitative feedback obtained from the participants during the discussion group.

1.1 Comprehensive Finger-Bow Force Measurements

This section comprises 12 figures encompassing all experimental conditions recorded during the study: three pedagogical exercises (static hold, *sostenuto* bowing, and a one-octave scale), two measured fingers (index finger and thumb), and two intentional finger-force levels (maximum and minimum force).

To illustrate the data processing pipeline and facilitate interpretation, each figure is systematically structured into three subpanels:

- (a) Raw Participants: Raw force data per participant, presented in the normalised scale (0–100) exactly as they observed it via the real-time graphical interface during the recordings.
- (b) Raw Statistics: The aggregated statistical data across all five participants, showing the mean signal alongside a shaded area representing the standard deviation (± SD) in the normalised scale.
- (c) Absolute Force: The processed force measurements converted to Newtons (N). To preserve the perceptual character of human intensity, these values are presented on a logarithmic scale, featuring the raw individual lines and the overlapping bold mean signal.

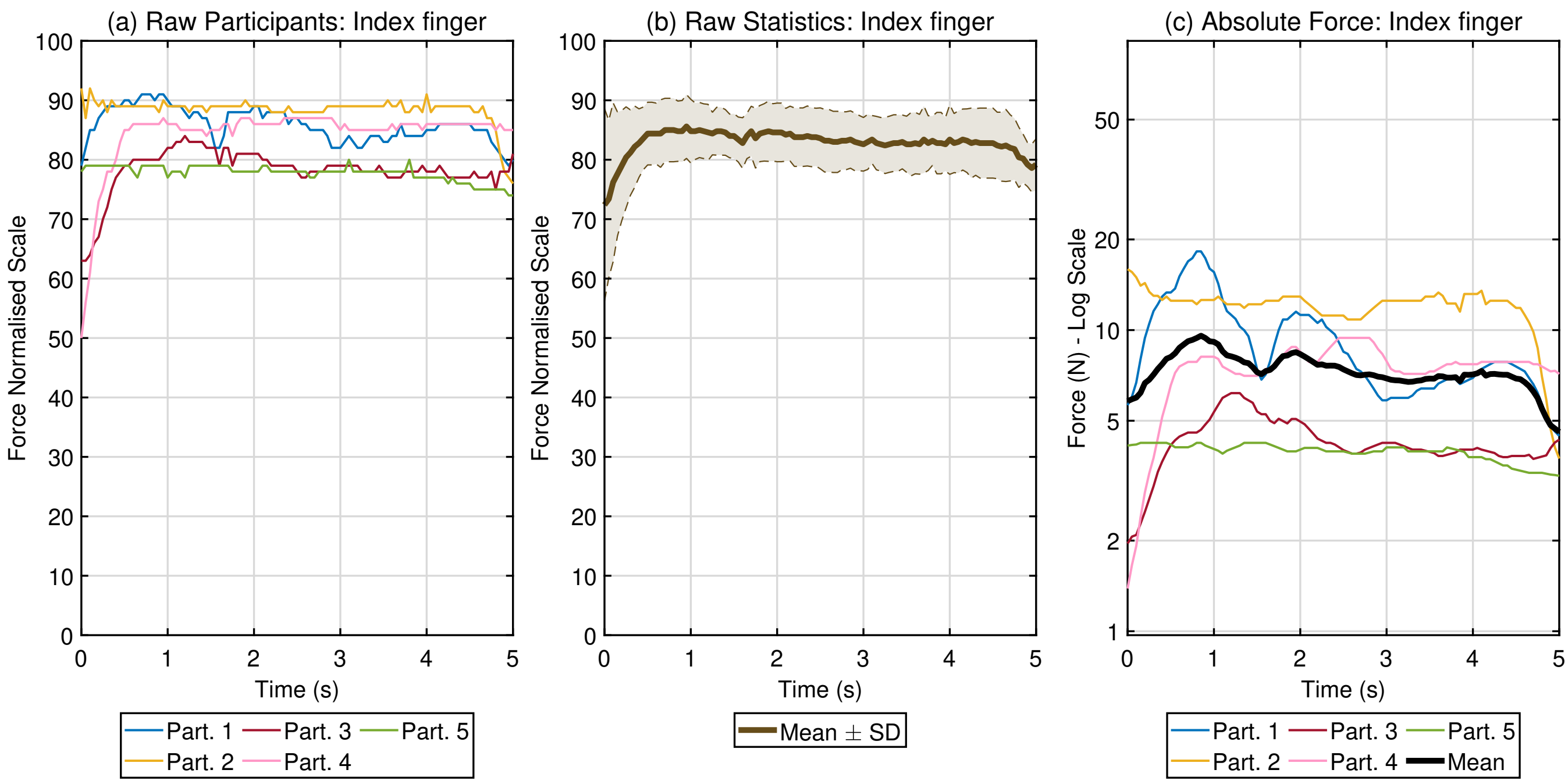

Figure S1. Force measurements for Exercise 1 (static bow hold), Index finger, Maximum force

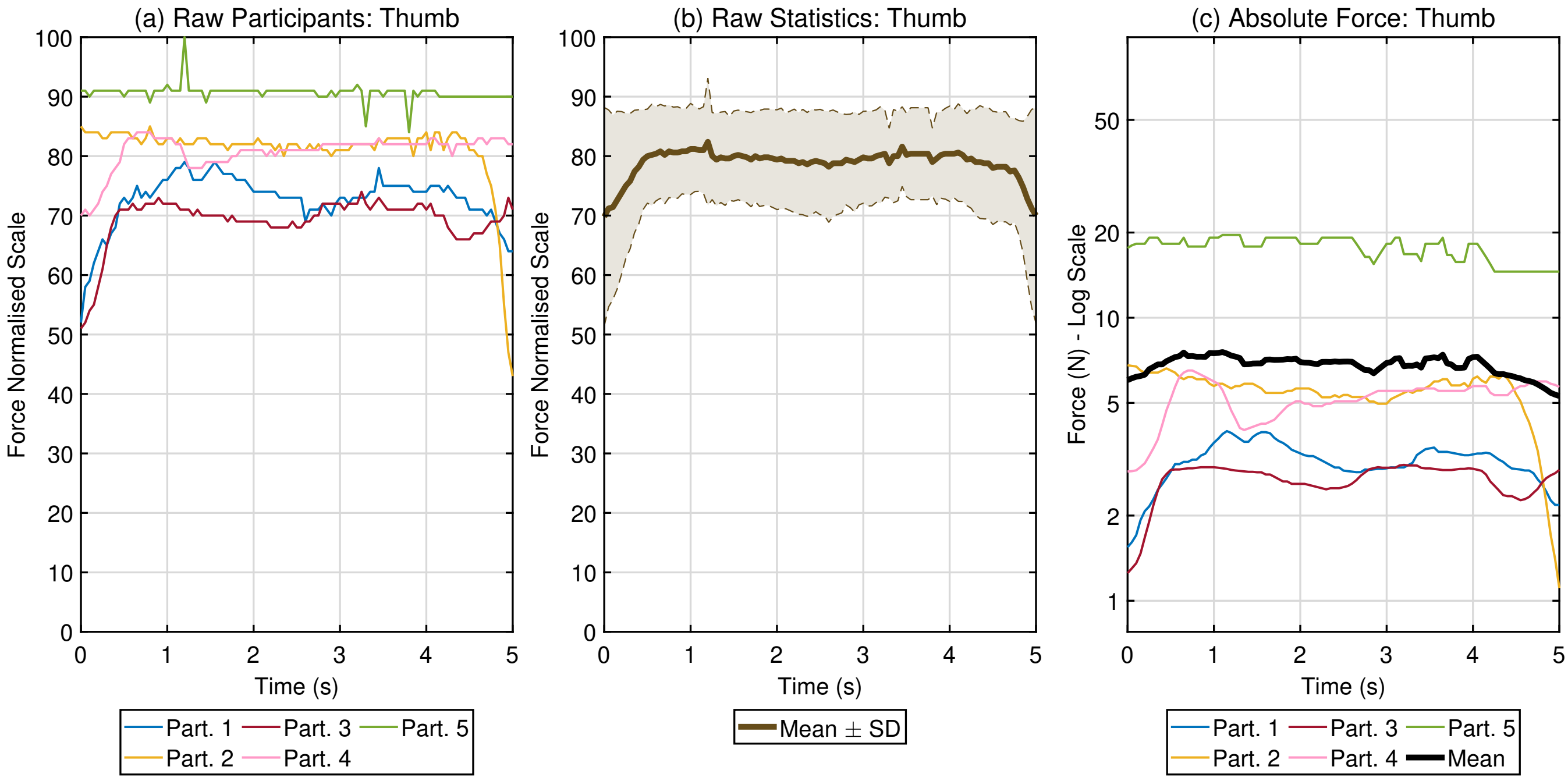

Figure S2. Force measurements for Exercise 1 (static bow hold), Thumb, Maximum force

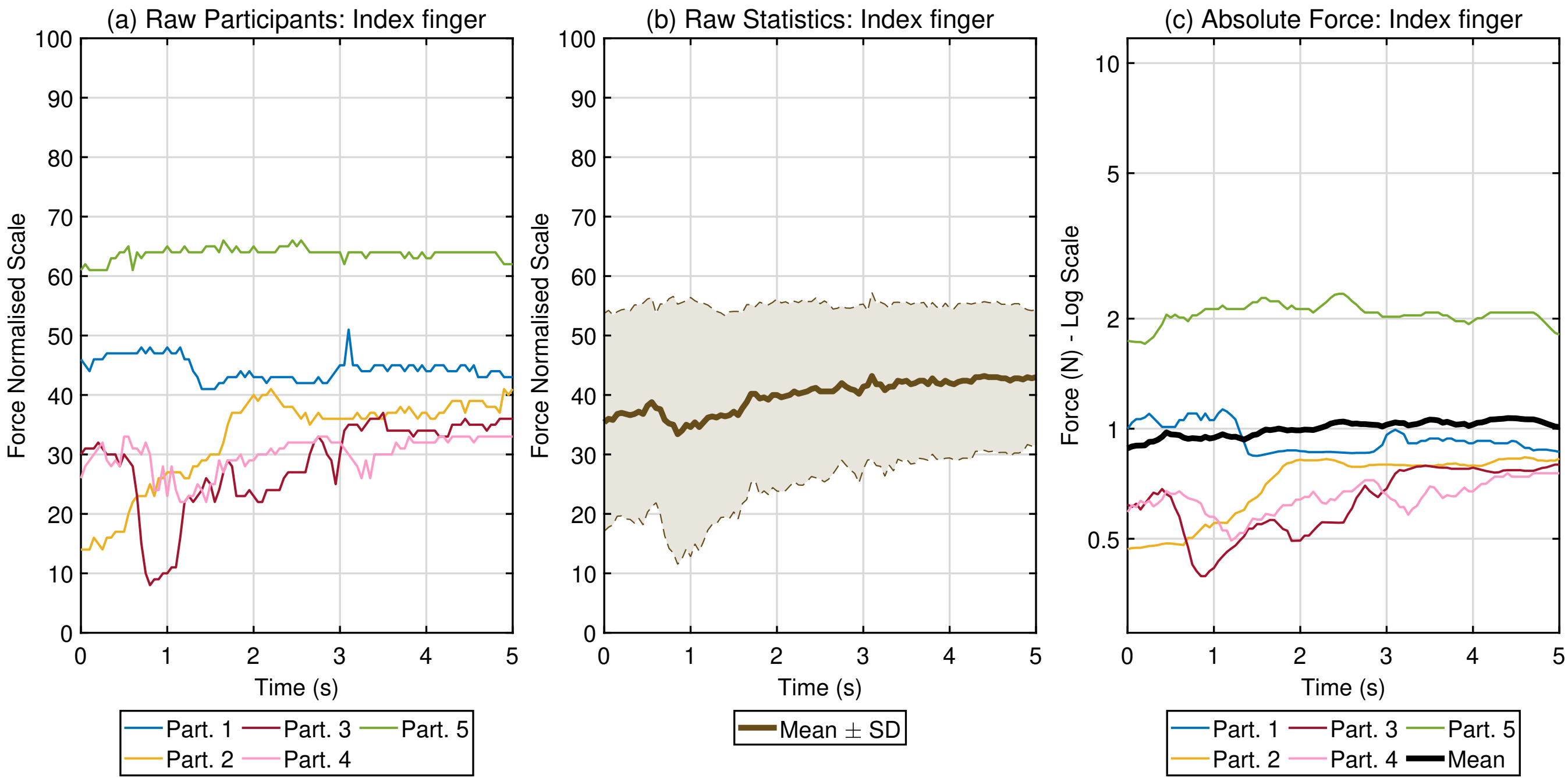

Figure S3. Force measurements for Exercise 1 (static bow hold), Index finger, Minimum force

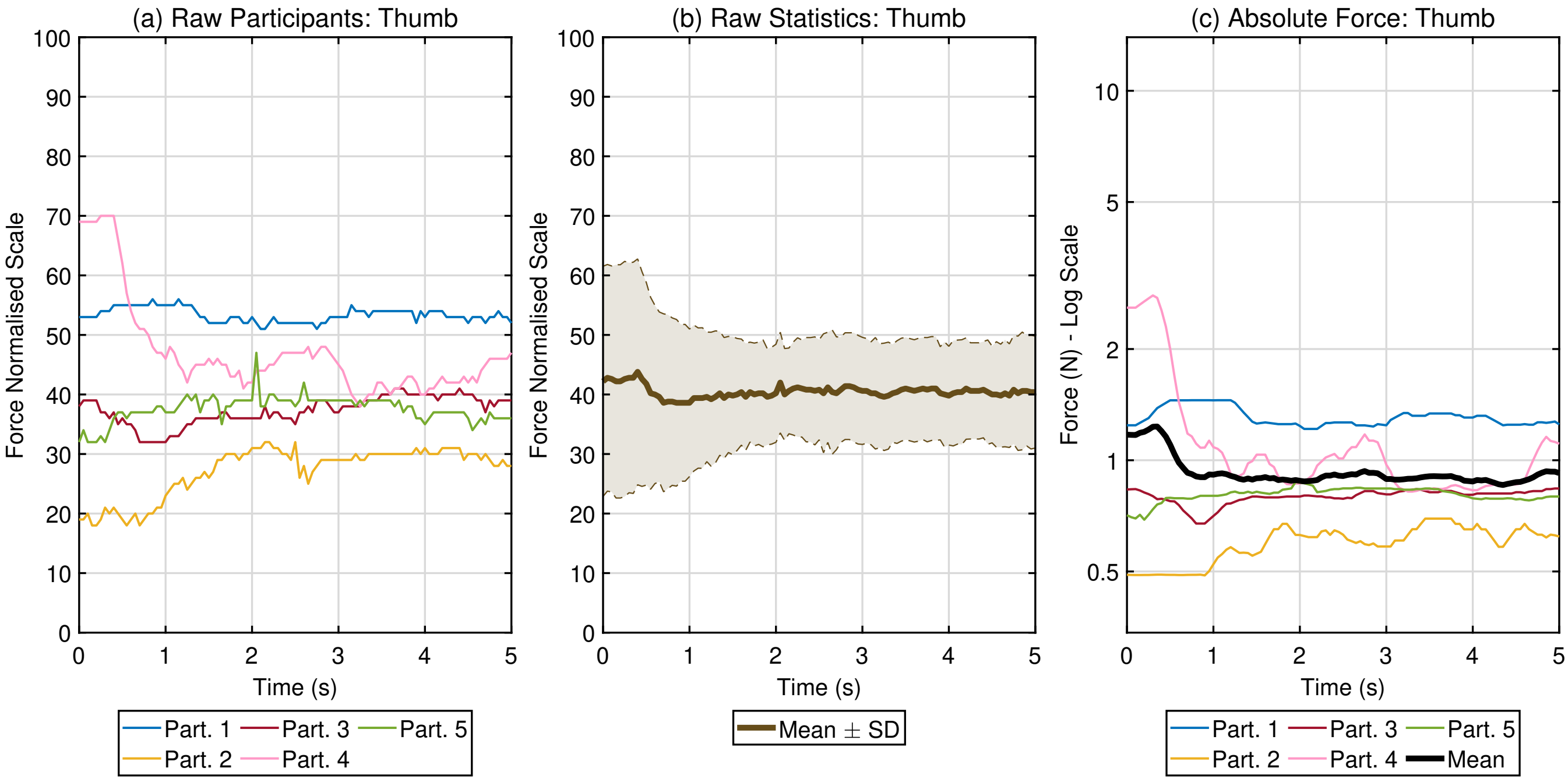

Figure S4. Force measurements for Exercise 1 (static bow hold), Thumb, Minimum force

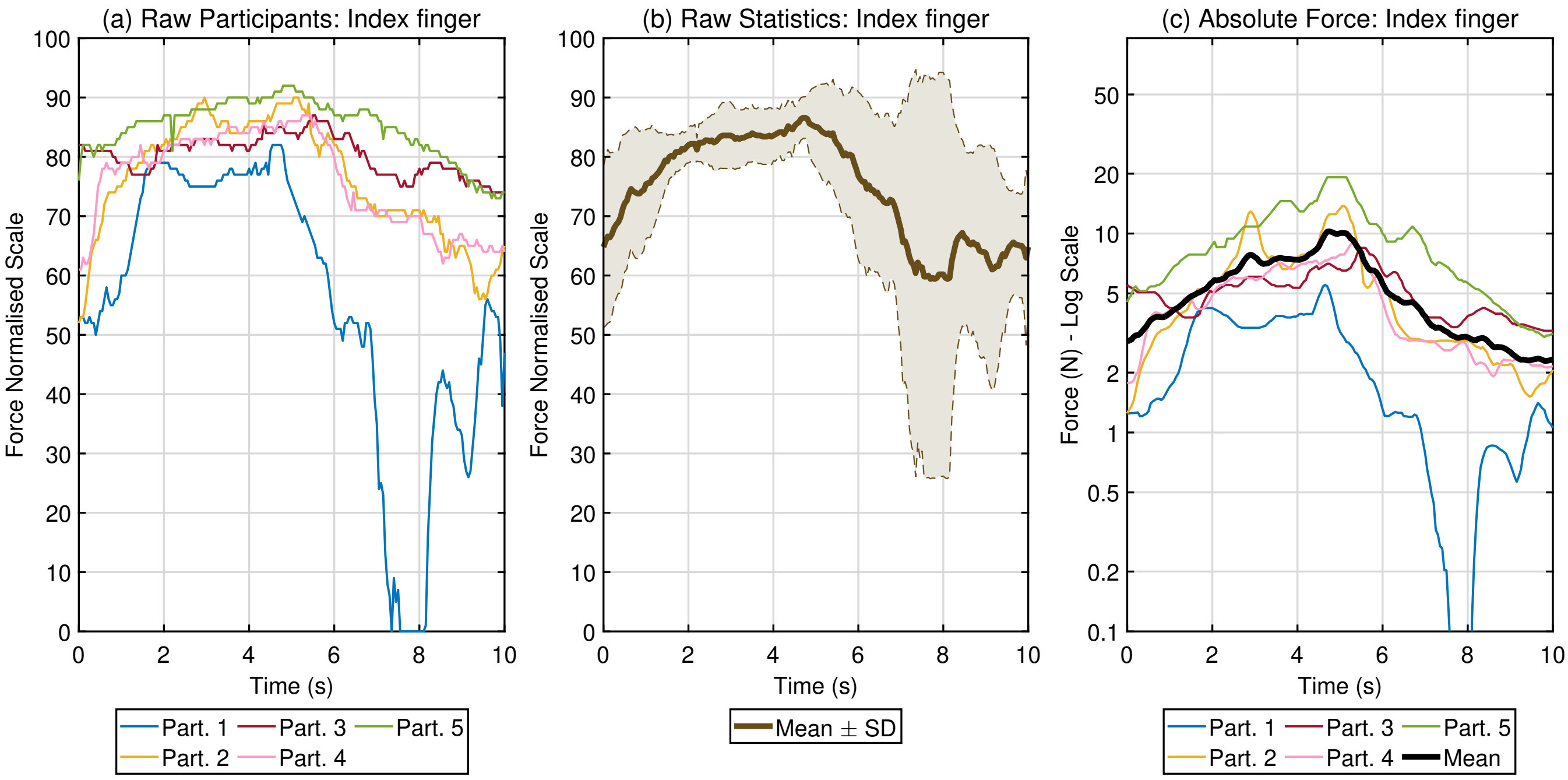

Figure S5. Force measurements for Exercise 2 (*sostenuto* bowing), Index finger, Maximum force

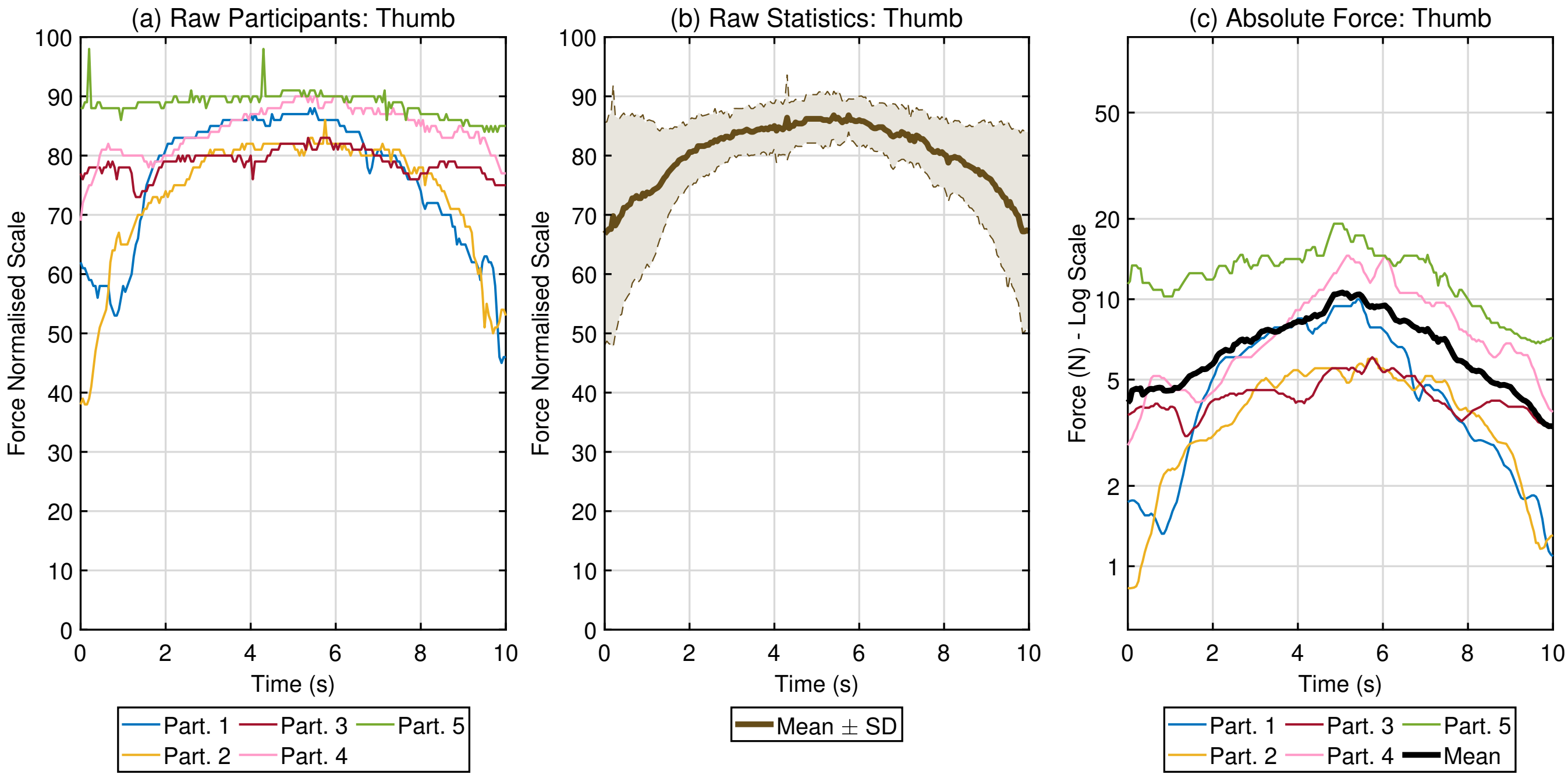

Figure S6. Force measurements for Exercise 2 (*sostenuto* bowing), Thumb, Maximum force

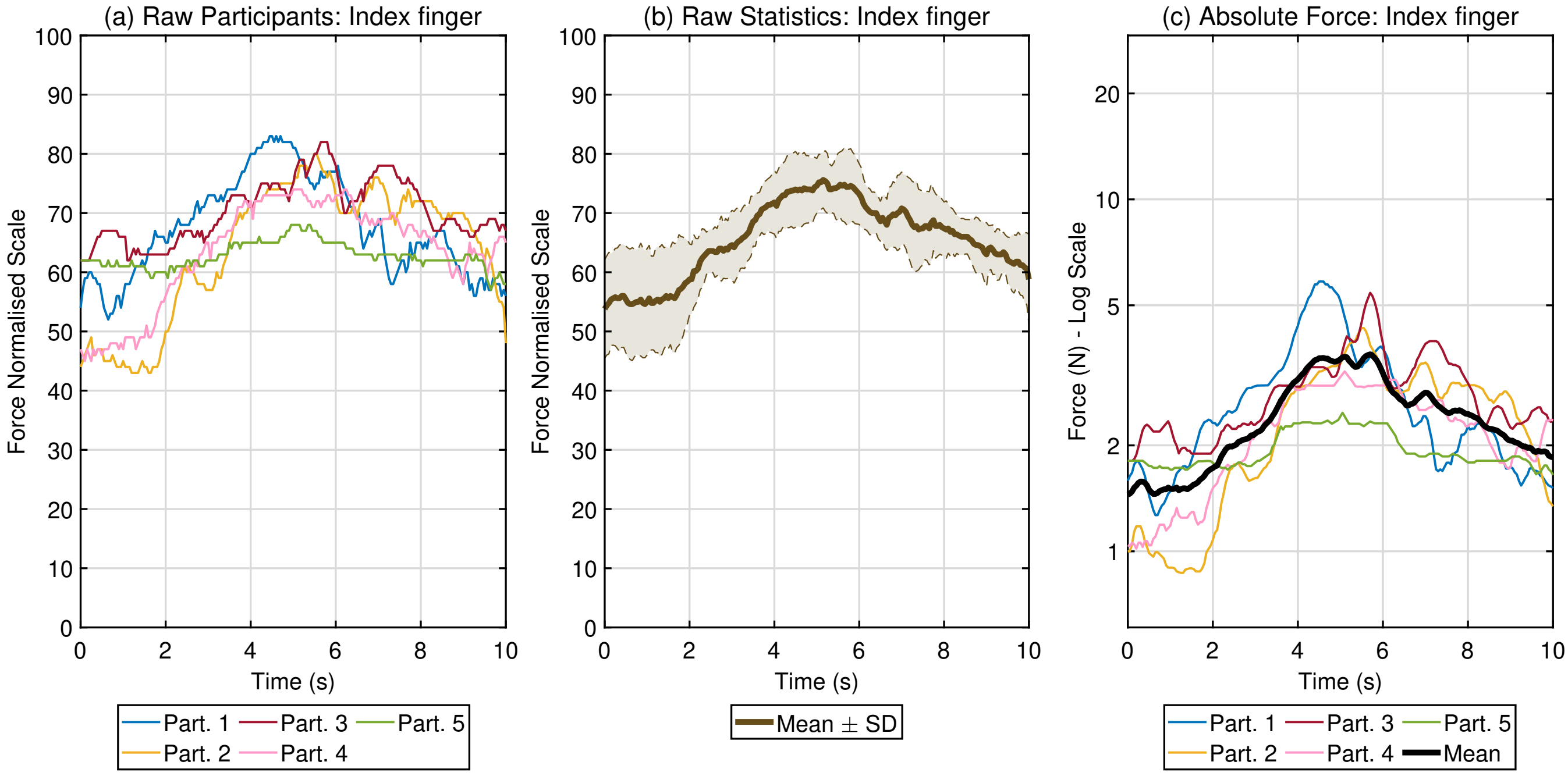

Figure S7. Force measurements for Exercise 2 (*sostenuto* bowing), Index finger, Minimum force

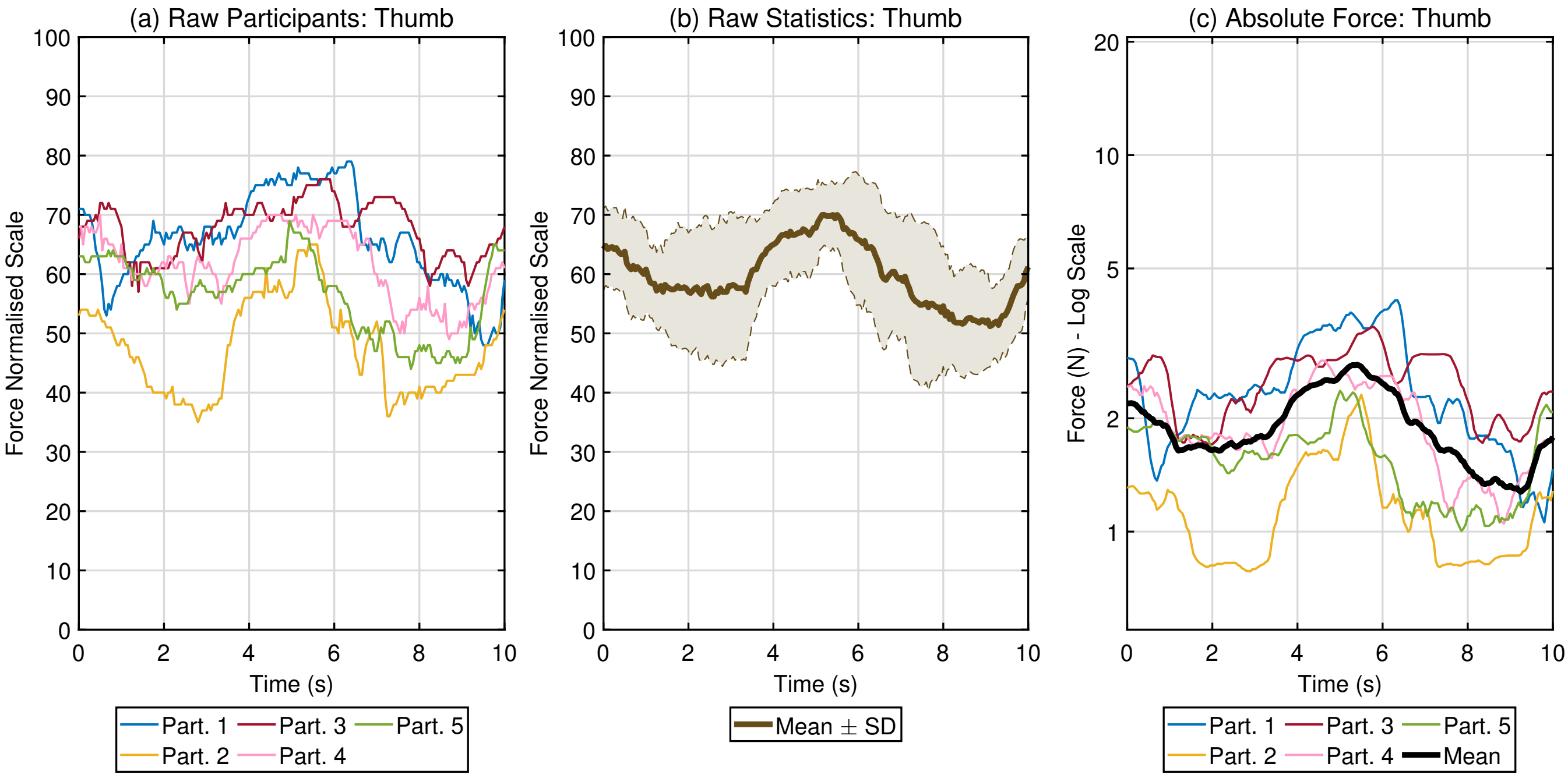

Figure S8. Force measurements for Exercise 2 (*sostenuto* bowing), Thumb, Minimum force

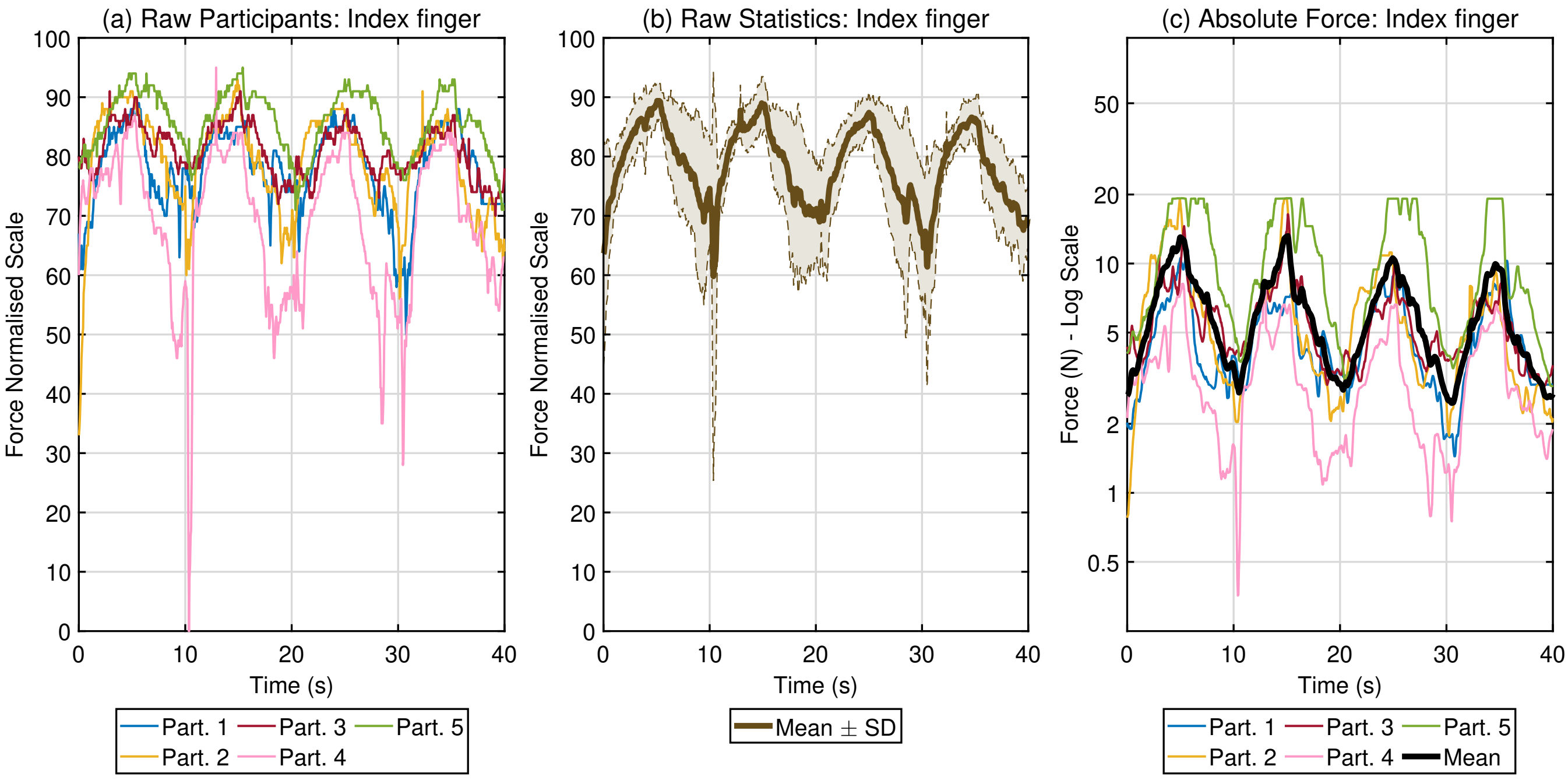

Figure S9. Force measurements for Exercise 3 (one-octave scale), Index finger, Maximum force

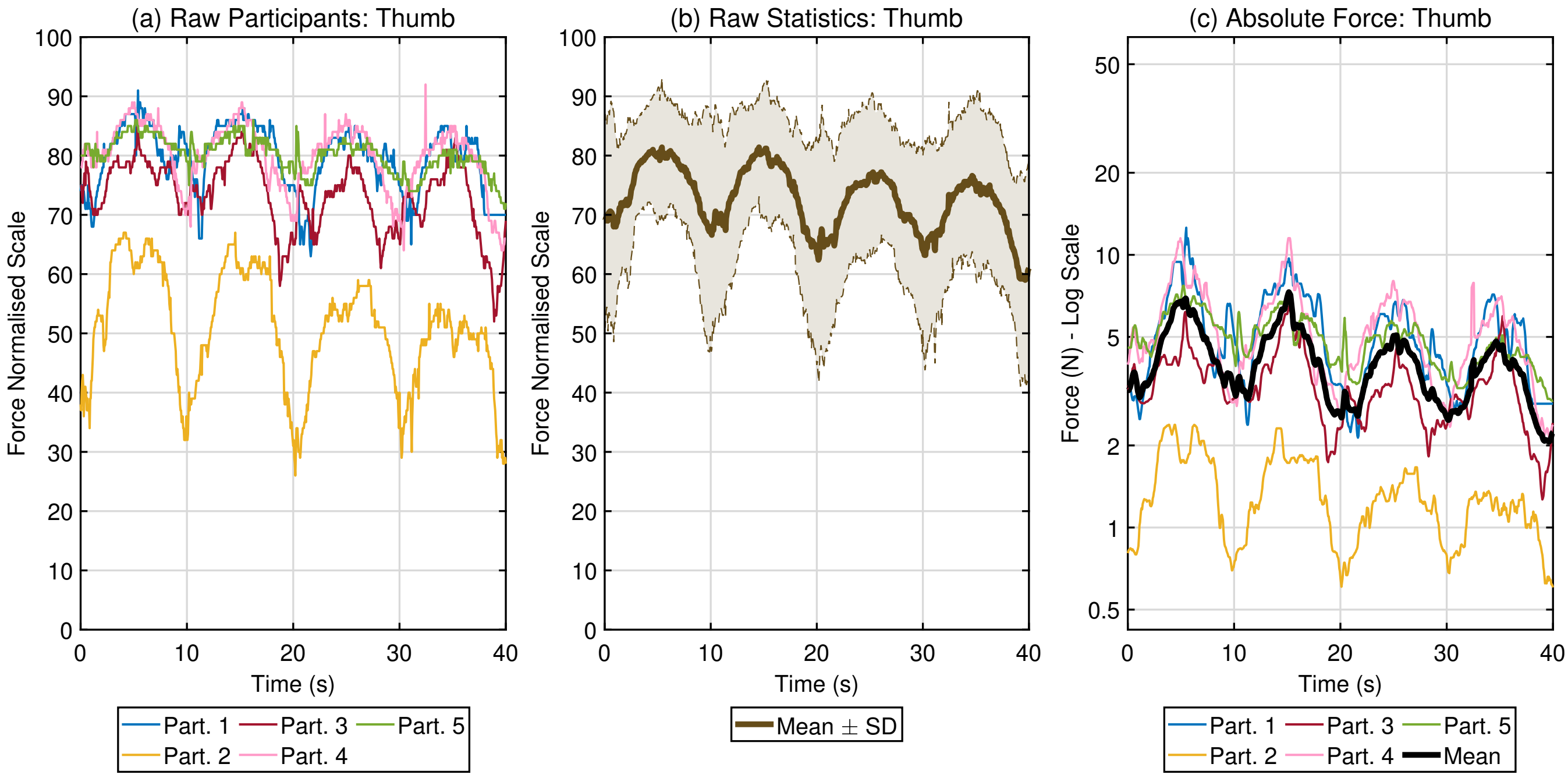

Figure S10. Force measurements for Exercise 3 (one-octave scale), Thumb, Maximum force

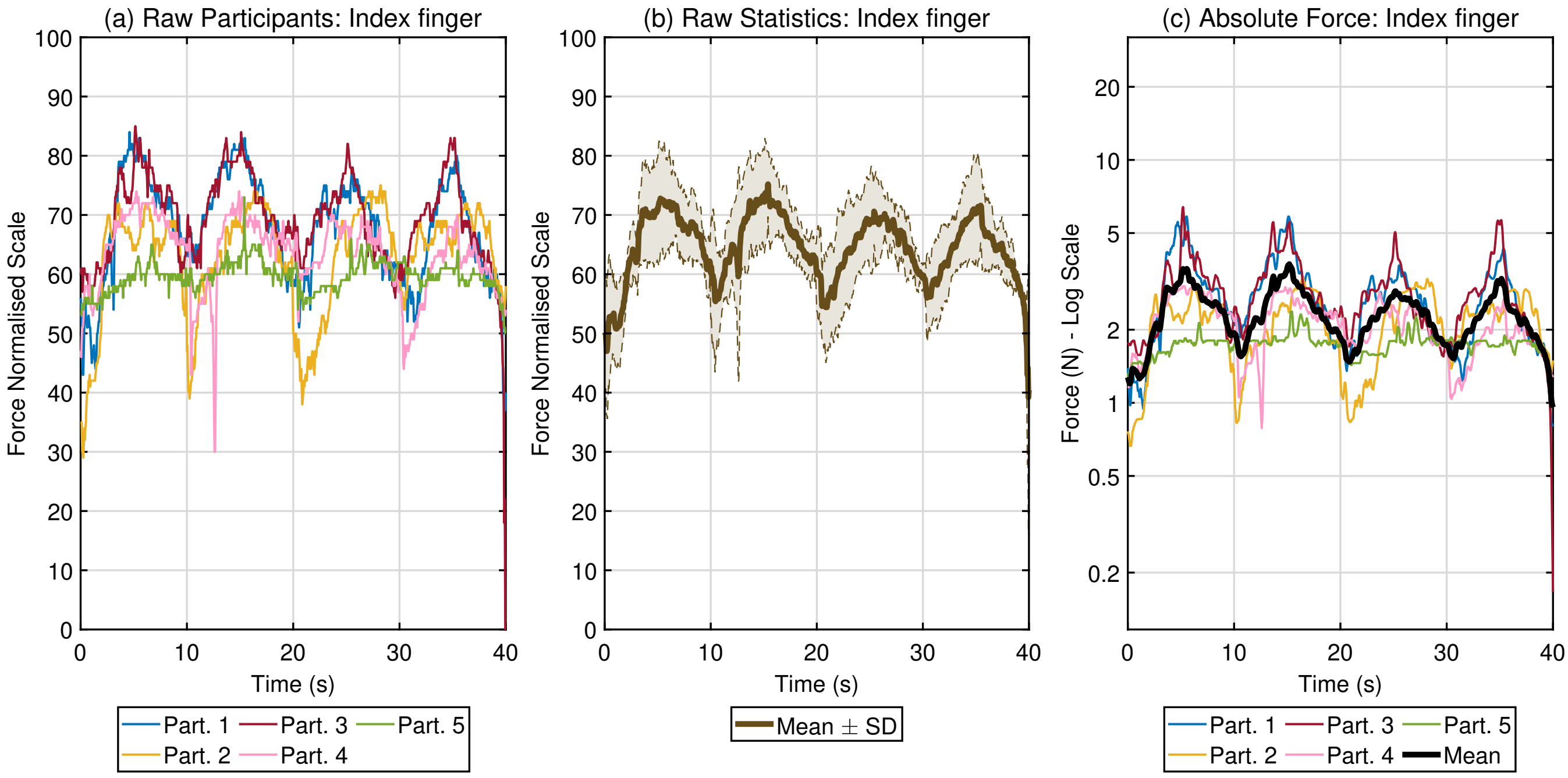

Figure S11. Force measurements for Exercise 3 (one-octave scale), Index finger, Minimum force

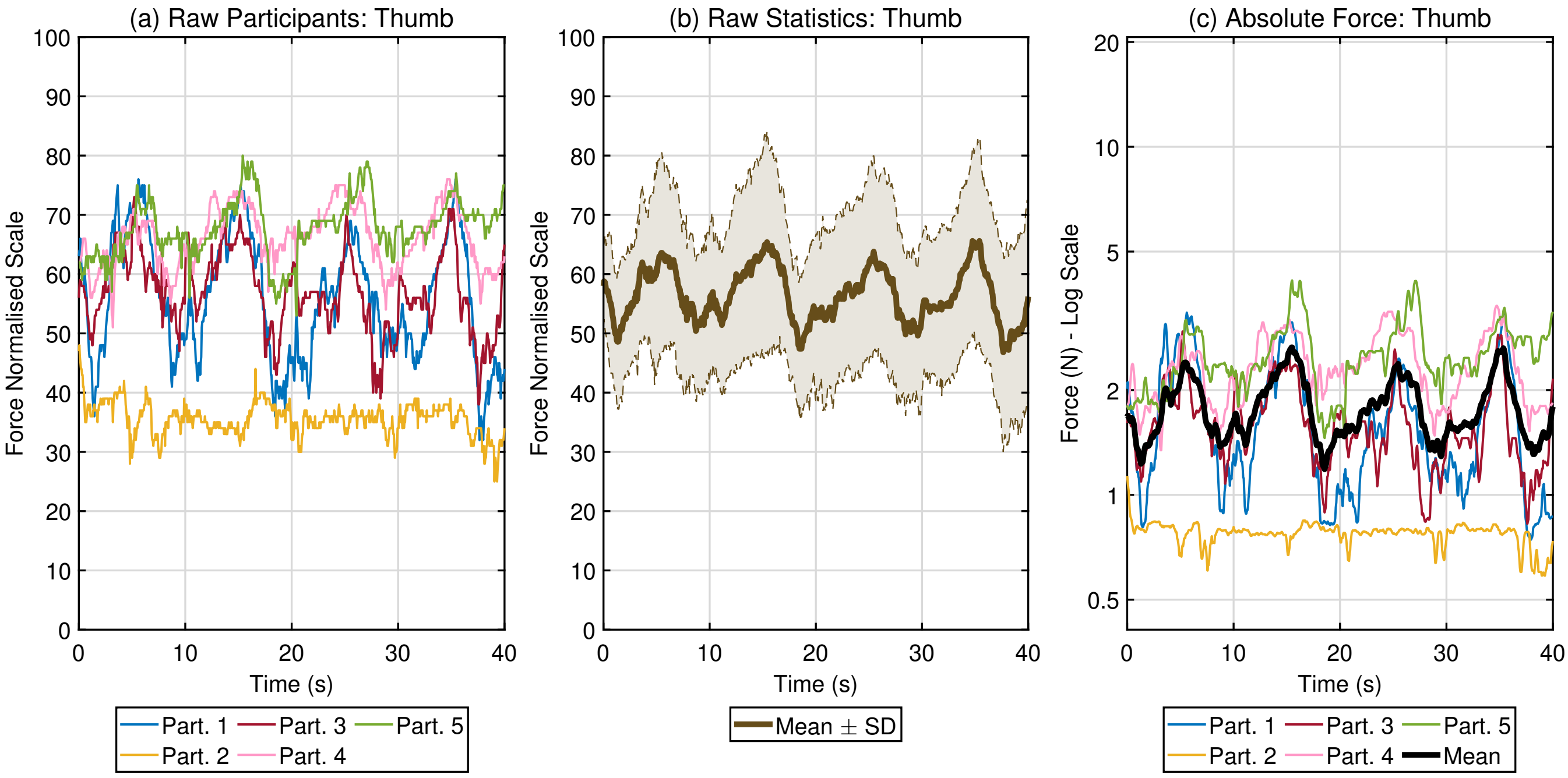

Figure S12. Force measurements for Exercise 3 (one-octave scale), Thumb, Minimum force

1.2 Comparative Representation: Linear vs. Logarithmic Force Scaling

While Section 1.1 presents force measurements on a logarithmic scale, this section provides an illustrative comparison displaying a single motor task across both linear and logarithmic domains. A standard linear scale provides an uncompressed quantitative view of physical loads, whereas compressing the vertical axis logarithmically mirrors the cellist's subjective tactile perception and aligns with the real-time classroom interface.

To demonstrate this dual perspective, Figure S13 is divided into three subpanels:

- (a) Raw Participants (Normalised Scale): The normalised 0–100 scale observed by the students in real time during the performance.
- (b) Absolute Force (Linear Scale): The processed force in Newtons displayed on a linear vertical axis, with the bold line indicating the mean across participants.
- (c) Absolute Force (Logarithmic Scale): The processed force measurements converted to Newtons (N). To preserve the perceptual character of human intensity, these values are presented on a logarithmic scale, featuring the raw individual lines and the overlapping bold mean signal.

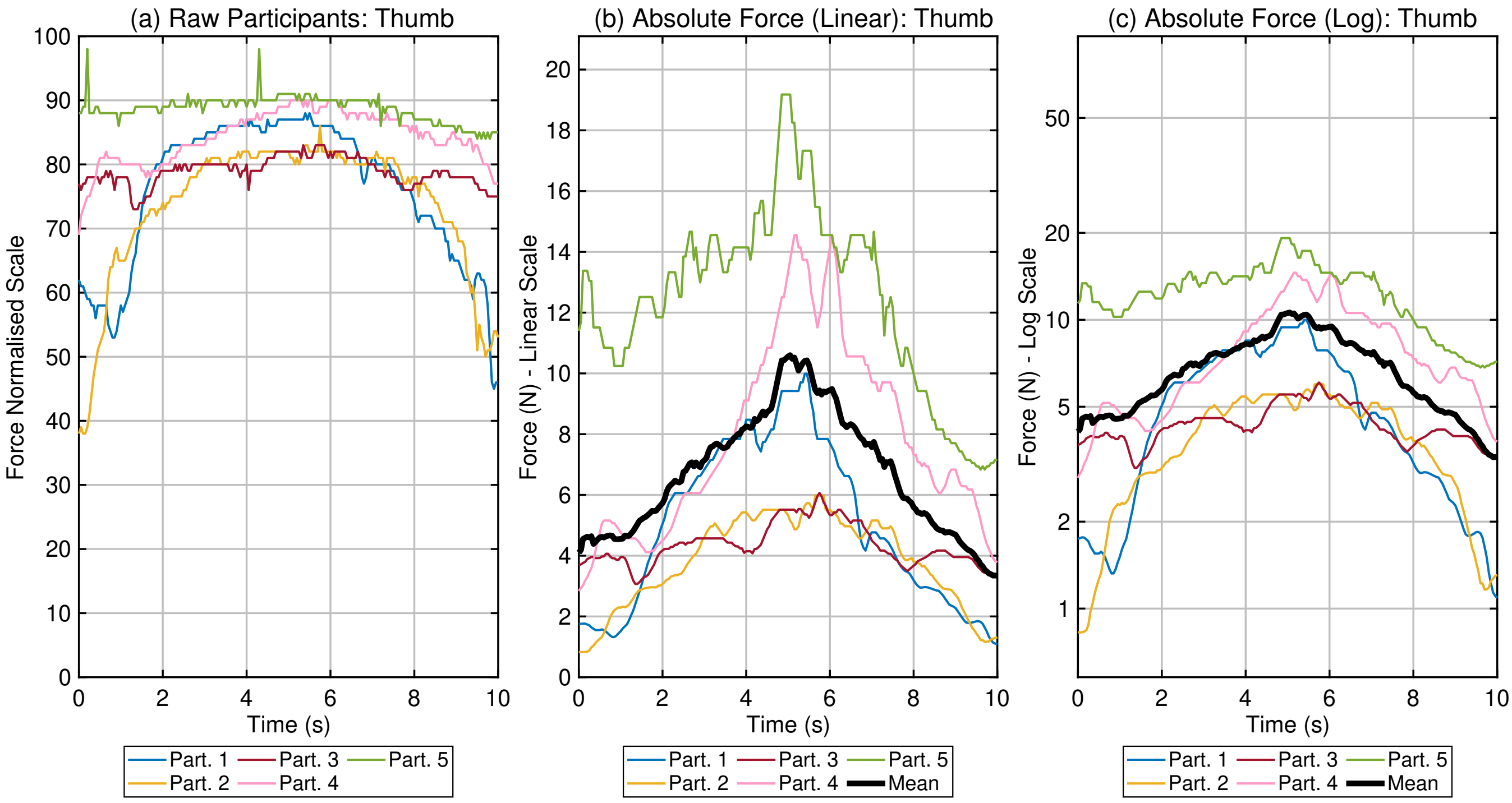

Figure S13. Force measurements for Exercise 2 (*sostenuto* bowing), Thumb, Maximum force

1.3 Qualitative Feedback from the Discussion Group

This section presents the detailed qualitative feedback collected during the post-intervention focus group. Table S1 categorises the participants' main observations into four thematic blocks: ergonomics and comfort, ease of interpreting the graphical interface, use as a tool for pedagogical self-monitoring, and awareness of the pressure exerted while playing. These responses complement the quantitative force measurements by providing direct insights into the usability, haptic experience, and perceived educational value of the sensorised glove from the students' perspective.

Table S1. Feedback from participants on the sensorised glove, organised in four categories and on the order in which they contributed to the discussion group.

Participant(s)	Main Observations
**Ergonomics and comfort**	
P4	Glove keeps middle, ring, and little fingers free; yet fear that sensors could move.
P1	Glove positively viewed as comfortable; yet unnatural sensation and limited hand mobility.
P5	Agreed with P1 on restricted movement.
P2	Sensors are too rigid and large.
P3	Comfortable; sensor rigidity did not affect exercise performance.
**Ease of interpreting the graphical interface**	
P2, P5	Very intuitive with separate coloured lines for thumb and index finger.
P1, P3, P4	Suggest limiting the visualisation to ”normal use” range with alerts for excessive/low pressure.
**Use as a tool for self-monitoring within a pedagogical process**	
P2	Great potential beyond bowing exercises; useful for personal practice and self-monitoring.
P5	Helps awareness of bow pressure and injury prevention.
P4	“Non-real” sensation may limit pedagogical effectiveness.
P3	Helps objectify subjective pressure sensation.
Moderator	Low-cost design essential for classroom/student use.
**Awareness of the pressure exerted while playing**	
P4	Noticed loss of index finger contact/pressure during bow changes.
P1	Index finger deforms into hook shape under pressure, losing weight.
P2	Helped maintain awareness of sustained bow weight over time.
P3	Pressure increases at bow tip to compensate for weight loss there.
